# Stochastic disease spreading and containment policies under state-dependent probabilities

**DOI:** 10.1007/s00199-023-01496-y

**Published:** 2023-04-12

**Authors:** Davide La Torre, Simone Marsiglio, Franklin Mendivil, Fabio Privileggi

**Affiliations:** 1 SKEMA Business School and Université Côte d’Azur, Sophia Antipolis Campus, Sophia Antipolis, France; 2 Department of Economics and Management, University of Pisa, Pisa, Italy; 3 Department of Mathematics and Statistics, Acadia University, Wolfville, Canada; 4 Department of Economics and Statistics “Cognetti de Martiis”, University of Turin, Turin, Italy

**Keywords:** Economic epidemiology, Invariant distribution, Optimal policy, State-dependent probability, C60, H50, I10

## Abstract

We analyze the role of disease containment policy in the form of treatment in a stochastic economic-epidemiological framework in which the probability of the occurrence of random shocks is state-dependent, namely it is related to the level of disease prevalence. Random shocks are associated with the diffusion of a new strain of the disease which affects both the number of infectives and the growth rate of infection, and the probability of such shocks realization may be either increasing or decreasing in the number of infectives. We determine the optimal policy and the steady state of such a stochastic framework, which is characterized by an invariant measure supported on strictly positive prevalence levels, suggesting that complete eradication is never a possible long run outcome where instead endemicity will prevail. Our results show that: (i) independently of the features of the state-dependent probabilities, treatment allows to shift leftward the support of the invariant measure; and (ii) the features of the state-dependent probabilities affect the shape and spread of the distribution of disease prevalence over its support, allowing for a steady state outcome characterized by a distribution alternatively highly concentrated over low prevalence levels or more spread out over a larger range of prevalence (possibly higher) levels.

✉ Simone Marsiglio
simone.marsiglio@unipi.it

Davide La Torre
davide.latorre@skema.edu

Franklin Mendivil
franklin.mendivil@acadiau.ca

Fabio Privileggi
fabio.privileggi@unito.it

**JEL Classification** C60 · H50 · I10

## 1 Introduction

Infectious diseases have historically played a major role in shaping the prospects of economic development both in industrialized and developing countries through a variety of microeconomic and macroeconomic channels (Acemoglu and Johnson 2007; Adda 2016; Bloom et al. 2022; Boucekkine et al. 2009; Lopez et al. 2006). The ongoing COVID-19 pandemics has shown more clearly than ever that understanding how to contain the spread of communicable diseases is essential not only to protect human lives but also to preserve economic prosperity (McKee and Stuckler 2020; World Bank 2020). The economic epidemiology literature has extensively discussed the role of disease containment policies, mainly in the form of pharmaceutical interventions (generally classified as either preventive or treatment measures), in both limiting the spread of epidemic diseases (Anderson et al. 2010; Federico et al. 2022; Goldman and Lightwood 2002; Gersovitz and Hammer 2004; Philipson 2000) and supporting economic activity (Fabbri et al. 2023; Goenka and Liu 2012, 2019; Goenka et al. 2014; La Torre et al. 2020). The issue has become even more popular following the COVID-19 outbreak, when a huge and growing number of works has analyzed from a normative perspective the optimal policy response to balance the economic and health trade-off involved in non-pharmaceutical interventions, such as social distancing, lockdowns and travel bans (Acemoglu et al. 2021; Alvarez et al. 2021; Eichenbaum et al. 2021; La Torre et al. 2021). Despite the high level of uncertainty associated with epidemic dynamics most of the studies have assumed that disease spreading is entirely deterministic, and very limited are those exploring the implications of stochasticity on the determination of the optimal containment policy (Federico and Ferrari 2021; Hong et al. 2021; Shevchenko et al. 2021). Federico and Ferrari (2021) analyze how randomness in the disease transmission rate as well as in the time horizon impact policymakers' optimal response. Hong et al. (2021) shows that accounting for stochasticity in disease transmission yields richer optimal mitigation strategies than those derived in deterministic contexts. Shevchenko et al. (2021) discuss how stochastic epidemic shocks affect economic and environmental conditions analyzing their impact on optimal climate change policies. In all these works the probability of shocks affecting disease spreading is constant and thus completely independent of the level of disease prevalence. This is a strong simplification of reality where prevalence determines the likelihood of epidemic-related shocks by influencing disease incidence and individuals' behavioral responses. Our paper tries contributing to this scant literature by exploring how the optimal containment policy is related to stochastic shocks under state-dependent probabilities, that is the probability of shock realization depends on disease prevalence. State-dependent probabilities are a straightforward generalization of constant probabilities which allow to account for the mutual relation between epidemic shocks and epidemic dynamics.

Specifically, we develop a stylized economic-epidemiological framework in which the social planner needs to choose the optimal mitigation policy to limit the spread of an infectious disease by determining the intensity of treatment measures, accounting for the effects of stochastic shocks. Random shocks are associated with the diffusion of a new strain of the disease, which affects disease prevalence both additively (by increasing the number of infectives) and multiplicatively (by modifying the growth rate of infection), and the probability of shocks realization is state-dependent. In particular, we allow the shocks probability to be either increasing or decreasing in the number of infectives to account for the eventual presence or absence of individuals' behavioral changes in an attempt to reduce their disease exposure, respectively. Such two alternative setups may be well suited to describe individuals' response to different types of infections (common diseases vs. potentially deadly diseases), and thus allow us to characterize from a normative perspective how the optimal policy may change according to the specific features of the epidemic threat. In this context we explicitly derive the optimal policy by solving in closed-form the Bellman equation associated with our stochastic framework with state-dependent probabilities. This allows us to analyze its stochastic steady state which is represented by an invariant distribution of disease prevalence, with support on strictly positive values meaning that complete eradication is never a possible outcome.

We also characterize how the properties of the invariant distribution are related to the characteristics (in terms of monotonicity and steepness) of the probability function. We derive two interesting sets of conclusions. First, the optimal policy is independent of the features of the state-dependent probabilities, and independently of them treatment allows to shift leftward the support of the invariant measure. This suggests that the disease containment efforts are effective in reducing the possible endemic prevalence levels associated with the steady state outcome. Second, the features of the state-dependent probabilities do matter as they affect the distribution of disease prevalence (in particular its shape and spread) over its support. In particular, their monotonicity property determines the shape of the invariant distribution: whenever the probability function is decreasing the steady state outcome is characterized by a skewed distribution highly concentrated over extremely low or high prevalence levels, while whenever it is increasing the disease outcome is associated with epidemic waves giving rise to a distribution more evenly spread out over a large range of prevalence (possibly higher) levels. The steepness property of the state-dependent probabilities instead determines where most of the mass is concentrated, that is whether the probability of low prevalence levels is higher or lower; however, the likelihood of low prevalence depends in a nontrivial way on the interactions between the monotonic and steepness characteristics of the state-dependent probability function. Moreover, we present a new result, more general than those discussed in extant literature (Mitra et al. 2003; Shmerkin 2014), determining sufficient conditions for the invariant measure to be either singular or absolutely continuous with respect to the Lebesgue measure, showing that this ultimately depends on the relative magnitude of the net infectivity rate, the extent to which the shock realization affects the net infectivity rate and susceptibles, and the weight attached to potential infections in the objective function. By extending the analysis to account for multiple shock realizations, we show that understanding the implications of the features of several state-dependent probabilities on the invariant distribution is more complicated as in this case we can no longer isolate the separate role played by each state-dependent probability function. Nevertheless, in a specific probabilities configuration allowing for comparability with our baseline framework, we can show that our conclusions regarding how the characteristics of the state-dependent probability function affect the steady state distribution apply also in more general contexts.

By introducing state-dependent probabilities in the determination of the optimal disease containment policy, our paper makes some interesting contributions in two different branches of the literature. With respect to the economic epidemiology literature (Goldman and Lightwood 2002; Gersovitz and Hammer 2004; Goenka et al. 2014; La Torre et al. 2020) which discusses that the economy may converge to a situation of eradication or endemicity according to the effectiveness of disease containment policies, we show that complete eradication is not possible and the steady state outcome is represented by an endemic state in which the distribution of disease prevalence may be more or less concentrated around lower or higher levels according to the characteristics of the shock probabilities. Methodologically, instead, we rely on the theory of iterated function systems with state-dependent probabilities to characterize the long run properties of the dynamic system associated with our economic-epidemiological framework. Iterated function systems (IFS) with constant probabilities have been extensively employed in economic applications to characterize the fractal properties of the steady state in stochastic optimal growth models (Montrucchio and Privileggi 1999; Mitra et al. 2003; Mitra and Privileggi 2009; La Torre et al., 2015), while iterated function systems with state-dependent probabilities (IFSSDP) have been frequently employed in the mathematics literature (Barnsley and Demko 1985; Stenflo 2002) and only seldom in economics (La Torre et al. 2019). Different from La Torre et al. (2019) who analyze how state-dependent probabilities affect the long run outcome in a purely dynamic context, we determine their implications on the optimal policy in a normative framework where the social planner specifically accounts for the role of state-dependent probabilities in its policy decisions. To the best of our knowledge, ours is the first attempt to address a stochastic dynamic optimization problem under state-dependent probabilities in economics.

The paper proceeds as follows. Section 2 introduces our stochastic epidemiological framework where random shocks associated with the diffusion of a new disease strain occur with state-dependent probabilities. Section 3 introduces our economic framework in which the social planner determines the optimal treatment policy accounting for the state-dependency of such probabilities. Section 4 explicitly derives the optimal solution discussing the role of the optimal policy in determining the steady state outcome and the role of state-dependent probabilities. Section 5 discusses the characteristics of the invariant measure in terms of singularity vs absolute continuity. Section 6 presents an extension of our baseline model to allow for shocks to take on any finite number of values. Section 7 as usual presents concluding remarks and highlights directions for future research. A brief review of the IFS theory and in particular on the theory of IFSSDP is presented in “Appendix A”, while the proofs of our main results are reported in “Appendices B and C”.

## 2 The epidemiological model

We start discussing the epidemiological context abstracting completely from containment policies in order to clarify our setup and the role of state-dependent probabilities. We develop a very simple framework to characterize the spread of a communicable disease, which may be either a common disease (i.e., the seasonal flu, the common cold) or a potentially deadly infection (i.e., SARS, COVID-19). Different from traditional epidemiological setups in which the interactions between different population groups (i.e., susceptibles and infectives) drive the epidemic dynamics, we focus only on the determinants of disease prevalence. In such a simplistic context we account for the uncertainty associated with infection diffusion by considering the role played by the arise of a new disease strain and by endogeneizing the likelihood with which random shocks occur assuming that the their probability is state-dependent.

The population size, which is constant and normalized to unity without loss of generality, $N \equiv 1$, is composed by healthy individuals who are susceptible to the disease, $S_t$, and the infectives who have already contracted the disease and can transmit it to susceptibles, $I_t$, thus at any moment in time we have that $1 = S_t + I_t$. We assume that the disease dynamics is described by the following equation:

$$I_{t+1} = \eta z_t \tilde{\Omega} I_t + \theta z_t S_t, \tag{1}$$

where $\tilde{\Omega} > 0$ measures the net infectivity rate (i.e., the infectivity rate net of the recovery rate) of the mainstream strain of the disease, $z_t$ denotes random shocks that can take one of two values, $r_1$ or $r_2$, such that $0 < r_1 < r_2$, while $\eta > 0$ and $\theta > 0$ quantify the extent to which the shock realization affects the net infectivity rate and susceptibles, respectively. The equation above states that the dynamics of disease prevalence crucially depends on the biological features of the disease ($\tilde{\Omega}$) and the realization of random shocks ($z_t$). Biological factors combined with the disease prevalence determine the disease incidence $\tilde{\Omega} I_t$ which characterizes the pace of disease diffusion in the presence of only one strain of the disease. The random shock term captures the twofold impact of a new disease strain on the evolution of the disease. (i) A new strain is discovered when a susceptible individual is found to be infected with a genetic variant of the microorganism (i.e., a virus or bacterium) causing the infectious disease. Thus, the diffusion of a new strain gives rise to some new infections not related to the single-strain disease incidence, captured by the additive term $+z_t S_t$. (ii) A new strain is characterized by different infectivity and recovery rates with respect to the original strain of the disease, such that the biological disease parameters change from one strain to the next. Thus, with the origin of a new strain the average biological parameters of the disease between strains change, such that the net infectivity rate may become higher or lower following the discovery of a new strain, captured by the multiplicative term $z_t \tilde{\Omega} I_t$. Such two effects associated with the diffusion of a new disease strain are weighted by parameters $\eta$ and $\theta$, which measure the impact generated on the multiplicative (i.e., disease incidence) and additive (i.e., new infections) terms in the equation above. By exploiting the fact that $S_t = 1 - I_t$ and, under the assumption that $\eta \tilde{\Omega} > \theta$, by defining $\Omega = \eta \tilde{\Omega} - \theta > 0$ as the overall infectivity rate (i.e., the net infectivity rate adjusted for the effects of shocks both on disease incidence and susceptibles), we can rewrite the dynamics in (1) as follows:

$$I_{t+1} = \Omega z_t I_t + \theta z_t, \tag{2}$$

which clearly shows that disease prevalence ultimately depends on disease incidence, the realization of random shocks, biological factors of the disease and the intensity with which shocks affect incidence and induce new infections. Despite the linear structure of (2), the additive term characterizing the new infections generated by the diffusion of a novel disease strain allows our model above to give rise to epidemic dynamics similar to those occurring in traditional mathematical epidemiology frameworks. Indeed, a typical implication of mathematical epidemiology settings which drives disease spreading and is fully restored in our model is that the growth rate of infection is positive at zero prevalence and monotonically decreases with disease prevalence converging to a negative value at full prevalence. The main peculiarity of our setup deals with the magnitude of the infection growth rate, which turns out to be infinitely large at zero prevalence.[1] Such a difference is due to the effects of random shocks: since the diffusion of a new disease strain affects prevalence by acting on susceptibles, the shock realization generates a larger effect on the infection growth rate whenever the disease is close to eradication, which thus will never be achieved because new disease strains will continually arise giving rise to new epidemic waves. Different from traditional mathematical epidemiology models in which diseases prevalence shows monotonic dynamics, as we shall clarify later, our setup can give rise to a broad variety of possible outcomes including non-monotonic dynamics, and random shocks in this context play a key role.

In particular, in our framework the probability of the realization of random shocks is not constant but state-dependent, that is it depends on the level of disease prevalence $p(I_t)$. Specifically, $\{z_t\}_{t=0}^{\infty}$ is a Bernoulli process such that at each date $t$:

$$z_t = \begin{cases} r_1 & \text{with probability } p\left(I_t\right) \\ r_2 & \text{with probability } 1 - p\left(I_t\right) \end{cases}, \tag{3}$$

where either $p' < 0$, that is the probability that the smaller shock value, $r_1$, (larger shock value $r_2$) is decreasing (increasing) in the number of infectives, or $p' > 0$, that is the probability that the smaller shock value, $r_1$, (larger shock value $r_2$) is increasing (decreasing) in the number of infectives. The former case represents a situation in which individuals do not automatically implement behavioral changes in response to increases in disease prevalence, and this in turn expands the spread of the disease

[1] More precisely, in our model the infection growth rate is given by $\frac{I_{t+1}-I_t}{I_t} = \frac{\Omega z_t I_t + \theta z_t - I_t}{I_t} = \Omega z_t - 1 + \frac{\theta z_t}{I_t}$, which diverges to $+\infty$ for $I_t \to 0^+$ and converges to $\Omega z_t - 1 + \theta z_t = \eta \tilde{\Omega} z_t - 1$ as $I_t \to 1^-$, where $\eta \tilde{\Omega} z_t - 1 \leq 0$ provided that condition (8) which we shall introduce later holds true. Similarly, the deterministic epidemiological dynamic in a standard SIS framework, which read as $I_{t+1} = (1-\delta) I_t + \alpha S_t I_t = (1-\delta) I_t + \alpha (1 - I_t) I_t$ where $\alpha > 0$ and $\delta > 0$ denote the infectivity and recovery rates respectively, shows an infection growth rate given by $\frac{I_{t+1}-I_t}{I_t} = \frac{(1-\delta)I_t + \alpha(1-I_t)I_t - I_t}{I_t} = \alpha - \delta - \alpha I_t$, which, provided that the condition $\alpha > \delta$ ensuring disease spreading (i.e., the basic reproduction number is larger than unity) holds true, is positive and finite for $I_t \to 0^+$ and converges to the negative value $-\delta < 0$ as $I_t \to 1^-$.

and thus also the eventual diffusion of a new strain. The latter case instead describes a situation in which individuals do automatically implement behavioral changes in response to increases in disease prevalence by reducing their possible exposure to the disease and this in turn limits the spread of the disease and thus also the likelihood of diffusion of a new strain. We believe that both scenarios are realistic conceptualizations of how a disease may spread following individuals' behavioral response, because such a response may largely depend on the biological features of specific diseases. For example, when dealing with common diseases (such as the seasonal flu) individuals rarely implement behavioral changes to limit their exposure thus the $p' < 0$ case may apply, while when dealing with potentially deadly diseases (such as COVID-19) behavioral changes may become predominant thus the $p' > 0$ case may apply instead. In the following we shall consider both scenarios and analyze how the features of the probability function $p(\cdot)$ may affect our conclusions.

In the traditional compartmental models employed in mathematical epidemiology the presence of a new disease strain is generally characterized by adding a new group of infectives in order to keep track of the number of individuals infected by the different disease strains (Martcheva 2015; Meehan et al. 2018). However, as we have learned from the ongoing COVID-19 epidemic, the microorganism causing the disease may mutate frequently; thus, in reality it is often not possible to distinguish infectives according to the variant of the disease they have been affected by. In order to account for this issue, our setup adds together all the individuals infected by different variants of the pathogen in a unique group of infectives. In particular equation (2) describes the evolution of the prevalence level of the disease accounting for all its existing strains based on the idea that there exists some universality in the features of epidemic dynamics independently of the specific epidemiological model underlying disease spreading (i.e., SI, SIS, SIR, SIRS...). A similar setup is frequently used in empirical applications to perform estimation and forecasting of the evolution of the number of infectives without specifying a particular epidemiological model, since often the only data available are those related to disease prevalence (Wang et al. 2021; Zakharov et al. 2020).

The idea of modeling epidemic outcomes in stochastic contexts without relying on traditional compartmental frameworks has been employed also in other works both in economics and epidemiology (Chakraborty et al. 2010; Ming et al. 2016). For example, even if not relying on mathematical epidemiology settings, Chakraborty et al. (2010) show that it may be possible to restore some of their epidemiological features by requiring the (exogenous) probability infection function to satisfy particular properties. Our approach is similar to theirs in the sense that the characteristics of the (endogenous) state-dependent probability function will allow us to enrich epidemic dynamics in order to give rise not only to results qualitatively similar to those traditionally discussed in mathematical epidemiology but also to a broader variety of possible outcomes. Indeed, in our setting the overall infectivity rate determines the growth factor of infection and if this is large enough disease prevalence will tend to increase over time, while if it is small prevalence will tend to decrease. By affecting the magnitude of such a growth factor, the state dependency of shocks realization may give rise to periods of positive and negative prevalence growth, resulting eventually in the occurrence of different disease waves. Specifically, in the $p' > 0$ case when prevalence is low the probability of the larger shock value is high and this tends to increase prevalence giving rise to an expansionary period of infection, but as prevalence increases also the probability of the smaller shock value rises and this tends to lower prevalence giving rise to a contractionary period of infection. Overall, periods of growing and shrinking prevalence may alternate one another over time characterizing multiple epidemic waves. In the $p' < 0$ case instead when prevalence is low the probability of the larger shock value is low and this tends to decrease prevalence giving rise to a contractionary period of infection, deterring the possibility of fast infection growth. Overall, periods of shrinking (or growing) prevalence may tend to persist over time characterizing monotonic epidemic dynamics. Note that if the probability of shocks were constant (i.e., $p' = 0$) such alternative outcomes would not be possible because the evolution of infection would resemble a random walk.

Despite the simplicity of (2) in describing epidemic dynamics, we believe that its ability to characterize endogenously the occurrence of periods characterized by monotonic epidemic dynamics or by multiple epidemic waves makes it a good benchmark to understand the working mechanisms of disease containment policies. Indeed, traditional mathematical epidemiological models cannot account endogenously for such alternative outcomes, as they are generally characterized by monotonic epidemic dynamics in which there is no room for multiple waves, and in order to explain alternate periods of growing and shrinking infections they usually rely on ad-hoc assumptions, such as the exogenous introduction of a periodic term to capture some cyclicality in disease transmission (Grassly and Fraser 2006; Jodar et al. 2008). Thus, the ability of our setup to describe within the same stylized framework monotonic epidemic dynamics and multiple epidemic waves, whose alternative occurrence depends on the specific features of the probability function, represents a novel approach to conceptualize disease spreading which may help us to better understand the role of containment policies in limiting the spread of infectious diseases. In particular we wish to clarify how the characteristics of the state-dependent probability function impacts epidemic dynamics and policymakers' optimal policy response.

A central role in our analysis needs thus to be placed on the features of the state-dependent probabilities. In this context, note that as the number of infectives $I_t$ in each period $t$ must lie in the interval $[0, 1]$, also the state-dependent probabilities have the same domain, that is, $p : [0, 1] \rightarrow [0, 1]$. In order to analyze explicitly the role of state-dependent probabilities, we introduce the following hyperbolic forms for $p(\cdot)$, defined for $I \in [0, 1]$:

$$p(I) = \frac{1}{BI^2 + 1} \quad \text{and} \quad 1 - p(I) = \frac{BI^2}{BI^2 + 1}, \quad \text{or} \tag{4}$$

$$p(I) = \frac{BI^2}{BI^2 + 1} \quad \text{and} \quad 1 - p(I) = \frac{1}{BI^2 + 1}, \tag{5}$$

where $B > 0$ is a parameter. Note that $p(I)$ and $1 - p(I)$ according to (4) actually have values in $\left[\frac{1}{B+1}, 1\right] \subset (0, 1]$ and $\left[0, \frac{B}{B+1}\right] \subset [0, 1)$ respectively, while $p(I)$ and $1 - p(I)$ according to (5) have values in $\left[0, \frac{B}{B+1}\right] \subset [0, 1)$ and $\left[\frac{1}{B+1}, 1\right] \subset (0, 1]$

respectively. Moreover, $p(I)$ in (4) is such that $p'(I) < 0$, so that the probability of the smaller shock value, $r_1$ (larger shock value, $r_2$) is decreasing (increasing) in the number of infectives; conversely, $p(I)$ in (5) is such that $p'(I) > 0$, so that the probability of the smaller shock value, $r_1$ (larger shock value, $r_2$), is increasing (decreasing) in the number of infectives. Therefore, (4) defines two (Lipschitz) continuous state-dependent probability functions satisfying $0 < p(I) \leq 1$ and $0 \leq 1 - p(I) < 1$ for all $0 \leq I \leq 1$, and (5) defines two (Lipschitz) continuous state-dependent probability functions satisfying $0 \leq p(I) < 1$ and $0 < 1 - p(I) \leq 1$ for all $0 \leq I \leq 1$.

The dynamics in (1) can be rewritten in terms of the following IFSSDP:

$$I_{t+1} = \begin{cases} r_1 \Omega I_t + \theta r_1 & \text{with probability } p(I_t) \\ r_2 \Omega I_t + \theta r_2 & \text{with probability } 1 - p(I_t), \end{cases} \tag{6}$$

which can be analyzed by relying on the IFS theory (see "Appendix A" for a brief review of the main tools and results needed for our analysis), which ensures the existence of a unique stationary distribution $\mu$ for such an IFSSDP supported on the interval $\left[I_1^{st}, I_2^{st}\right] \subset [0, 1]$, where the endpoints are the steady states of the two affine maps in (6) respectively:

$$I_1^{st} = \frac{\theta r_1}{1 - r_1 \Omega} \quad \text{and} \quad I_2^{st} = \frac{\theta r_2}{1 - r_2 \Omega}. \tag{7}$$

In order to keep the dynamics of $I_t$ defined by IFSSDP (6) inside the interval [0, 1] and rule out the trivial case $r_1 = 0$, the steady states of the above maps must satisfy the following parameter condition:

$$0 < r_1 < r_2 \leq \frac{1}{\eta \tilde{\Omega}}. \tag{8}$$

Note that (8) imposes an upper limit to the larger shock value in order to ensure that the dynamics in (6) remains trapped in a subset of [0, 1], and such an upper limit for $r_2$ can be either lower or larger than unity, depending on the values of the positive parameters $\eta$ and $\tilde{\Omega}$. This implies that the diffusion of a new disease strain may increase or decrease the growth rate of infection of the mainstream disease strain according to the specific parametrization, thus the total number of infectives could increase or decrease over time following the shock realizations. Moreover, note that, since the smaller shock value needs to be strictly larger than zero, the steady states of the maps in (7 ), which determines the left endpoint of the support of the invariant measure, do not include $I = 0$, which suggests that full eradication will never be possible. Since the diffusion of new disease strains affects additively epidemic dynamics, some new infectives will always be adding to the existing stock of infectives precluding the possibility for full eradication.

It is also interesting to observe that the characteristics of the probability function do not affect the support of the invariant measure, but they may affect the distribution of disease prevalence over its support. Unfortunately, characterizing this explicitly is not possible thus in the following we shall present some numerical example to illustrate the implications of different shapes of the probability function on the steady state distribution of disease prevalence. Specifically, we shall numerically approximate the time evolution of a given probability density according to the affine IFSSDP (6 ). To this purpose, in order to have a qualitative idea on what the limiting invariant measure may look like we apply a Maple algorithm[2] that approximates successive iterations of the Markov operator associated with our IFSSDP (see "Appendix A" for the definition of the Markov operator, given in (46)), based on Algorithm 1 in La Torre et al. (2019).

In the following numerical examples, we assume that the initial density is uniform and given by $\mu_0(I) \equiv \frac{1}{I_2^{st} - I_1^{st}}$, and we set the parameter values arbitrarily as follows:

$$\eta = 3, \qquad \tilde{\Omega} = \theta = 1, \qquad r_2 = \frac{1}{\eta\tilde{\Omega}} = \frac{1}{3}, \qquad r_1 = \frac{r_2}{2} = \frac{1}{6}, \tag{9}$$

such that $\Omega = \eta\tilde{\Omega} - \theta = 3 - 1 = 2$. We consider the two alternative values $B = \{3.571, 14.286\}$ in order to perform some comparative dynamics and to allow comparability with what we will present later when we determine the optimal disease containment policy. Indeed, as it will become clearer in Sect. 4, our goal is to obtain a closed-form solution for a planning problem and for this to be possible some parameter restrictions are required, and in particular the constant $B$ needs to take on one of the specific values we have considered in our parametrization. Figure 1 plots probabilities $p(I)$ (left panels) and $1 - p(I)$ (right panels) defined as in (4) for $B = 3.571$ (top panels) and $B = 14.286$ (bottom panels): clearly, $p(I)$ is decreasing in Fig. 1a, c while $1 - p(I)$ is increasing in Fig. 1b, d; the difference between the top and bottom figures is related to the more pronounced steepness in the bottom panels.

Under the parametrization in (9), our IFSSDP (6) reads as follows:

$$I_{t+1} = \begin{cases} \Omega r_1 I_t + r_1 = \frac{1}{3} I_t + \frac{1}{6} & \text{with probability } p(I_t) \\ \Omega r_2 I_t + r_2 = \frac{2}{3} I_t + \frac{1}{3} & \text{with probability } 1 - p(I_t), \end{cases} \tag{10}$$

and it has $\left[I_1^{st}, I_2^{st}\right] = [0.25, 1]$ as trapping interval (the fixed point of the upper map is 1 due to our choice on the larger shock to be exactly its admissible upper bound: $r_2 = \frac{1}{\eta\tilde{\Omega}}$). As the fixed point of the lower map, $I_1^{st} = 0.25$, is bounded away from 0, both $p(I_t)$ and $1 - p(I_t)$ are such that $0 < p(I_t) < 1$ and $0 < 1 - p(I_t) < 1$ for all $I \in [0.25, 1]$. Hence, the dynamics of $I_t$ will always remain trapped in the proper sub-interval $[0.25, 1] \subset [0, 1]$.

We consider first the scenario in which $p(I)$ is decreasing and specifically $p(I_t)$ takes the form in (4). The support of the invariant measure is [0.25, 1] (due to Corollary 1 in "Appendix A"); because the left endpoint of the trapping interval [0.25, 1] is bounded away from 0, the values of both $p(I)$ and $1 - p(I)$ will always be bounded away from 0 and 1 (as required by Theorem 3 in "Appendix A" to establish existence and uniqueness of the invariant measure). Figure 2 shows the initial uniform density $\mu_0(I) \equiv \frac{1}{I_2^{st} - I_1^{st}}$ (left panels), the 1st (mid panels) and 6th (right panels) iterations

[2] The detailed code is available upon request.

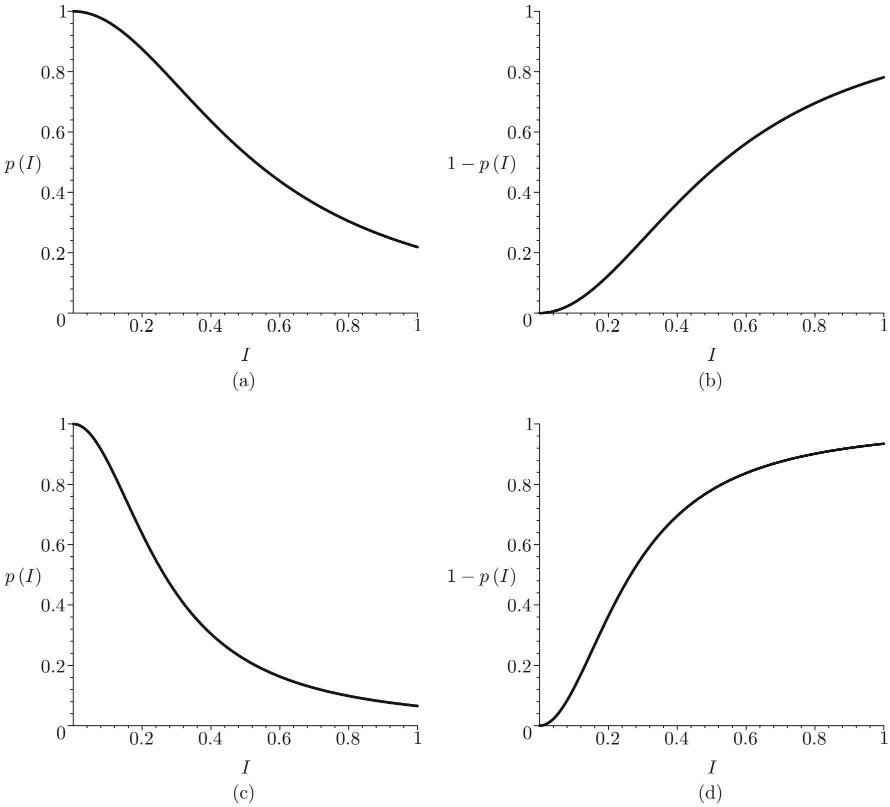

**Fig. 1** State-dependent probabilities, **a**, **c** $p\,(I) = \frac{1}{BI^2+1}$ and **b**, **d** $1 - p\,(I) = \frac{BI^2}{BI^2+1}$, associated to shocks $r_1 = \frac{1}{6}$ and $r_2 = \frac{1}{3}$ respectively, as defined in (4) for $B = 3.571$ (top) and $B = 14.286$ (bottom)

of our Maple algorithm for the IFSSDP (10) whenever $B = 3.571$ (top panels) or $B = 14.286$ (bottom panels). As convergence toward the unique invariant measure is geometric, i.e. very fast, Fig. 2c, f can be considered as good approximations of the invariant measure itself. As $\frac{1}{3} + \frac{1}{6} = \frac{1}{2} = \frac{2}{3}\frac{1}{4} + \frac{1}{3}$, the images of the two affine maps in (10) *almost* do not overlap, having in common the only point $\frac{1}{2}$ so that the invariant measure has the full interval [0.25, 1] as support. In the case of a small $B$, Fig. 2b shows that the IFSSDP concentrates a large probability mass of the uniform density in Fig. 2a close to the lower fixed point $I_1^{st} = 0.25$, and this process is being reinforced after each iteration so to obtain, after 6 iterations, Fig. 2c, in which the mass concentrated in the vicinity of $I_1^{st}$ has become predominant. In the case of a large $B$, Fig. 2e shows that the IFSSDP concentrates a large probability mass of the uniform density close to the higher fixed point $I_2^{st} = 1$, such that after 6 iterations in Fig. 2f larger mass is concentrated in the vicinity of $I_2^{st}$. Therefore, whenever $p(I_t)$ is decreasing, in the medium-long run the epidemic dynamics are characterized by a monotonic variation in infections. If $B$ is small (large) such a monotonic dynamic is associated with a reduction (increase) in infections which may increase only (also)

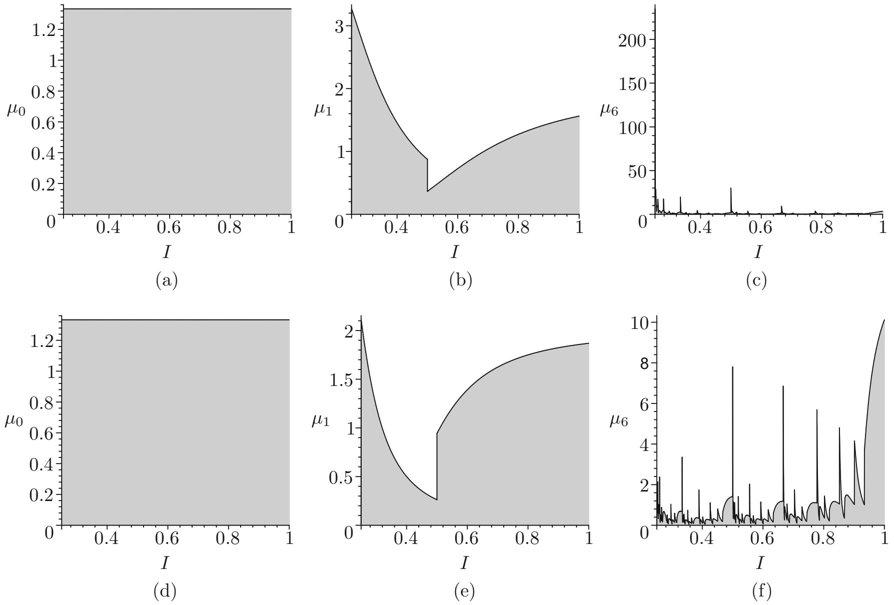

**Fig. 2** Initial uniform density over [0.25, 1] (left), 1st (mid) and 6th (right) iterations of our Algorithm to approximate the Markov operator (46) associated to the IFSSDP (10) whenever $p(I) = \frac{1}{BI^2+1}$ with $B = 3.571$ (top) or $B = 14.286$ (bottom)

because of the additive shock induced by the diffusion of a new disease strain, such that the level of disease prevalence tends to be concentrated to a large extent near the lower (upper) extreme of the support of the invariant measure and the steady state outcome is represented by an endemic state with low (high) prevalence.

We consider now the scenario in which $p(I)$ is increasing and specifically $p(I_t)$ takes the form in (5). Because the trapping interval is still [0.25, 1], again the values of both $p(I)$ and $1 - p(I)$ are bounded away from 0 and 1 (Theorem 3 in "Appendix A" still applies). Figure 3 shows the initial uniform density $\mu_0(I) \equiv \frac{1}{I_2^{st} - I_1^{st}}$ (left panels) and the 1st (mid panels) and 6th (right panels) iterations of our Maple algorithm for the IFSSDP (10) whenever $B = 3.571$ (top panels) or $B = 14.286$ (bottom panels). In the case of a small $B$, Fig. 3b shows that the IFSSDP concentrates a large probability mass of the uniform density in Fig. 3a around the interval [0.4, 0.5], Such a pattern, although scattered across all pre-fractals of the interval [0.25, 1] emerging after each iteration, is clearly preserved in the medium-long term approximation of the probability measure plotted in Fig. 3c. In the case of a large $B$, Fig. 3e shows that the IFSSDP concentrates a large probability mass of the uniform density in Fig. 3d to the left of 0.5, such that after 6 iterations in Fig. 3f a larger mass is concentrated close to the lower fixed point $I_1^{st} = 0.25$. Therefore, whenever $p(I_t)$ is increasing, in the medium-long run the epidemic dynamics are characterized by fluctuations in the level of infections, giving rise to multiple epidemic waves: the additive shocks combined with the higher incidence due to the diffusion of a new disease strain lead the number of infectives to continually rise and fall. If $B$ is small (large) such fluctuations are

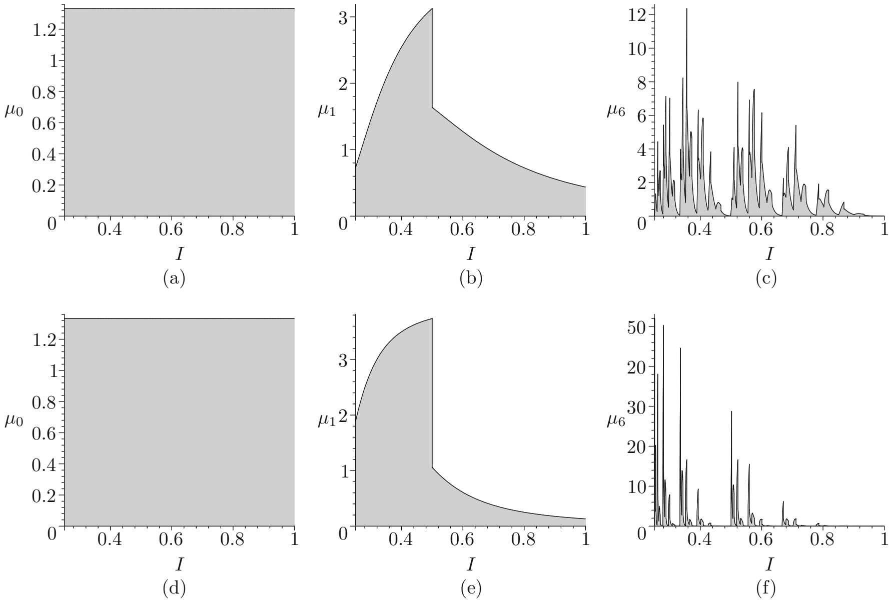

**Fig. 3** Initial uniform density over [0.25, 1] (left), 1st (mid) and 6th (right) iterations of our Algorithm to approximate the Markov operator (46) associated to the IFSSDP (10) whenever $p(I) = \frac{BI^2}{BI^2+1}$ with $B = 3.571$ (top) or $B = 14.286$ (bottom)

associated on average with a larger (lower) number of infections, such that the level of disease prevalence tends to be dispersed but more densely concentrated toward to upper (lower) extreme of the support of the invariant measure and the steady state outcome is represented by an endemic state with diffuse prevalence.

By comparing Figs. 2 and 3, we can observe that, despite the fact that the support of the invariant measure does not change with the characteristics of the probability function, the properties of the state-dependent probability (both in terms of the sign and size of its first derivative) critically determine the distribution of disease prevalence over its support. The sign of the derivative of the probability function determines the shape of the invariant distribution, and in particular whether this tends to be skewed or more symmetric over its support. Whenever $p' < 0$ the distribution tends to be more skewed toward one of the two extremes of the support (Fig. 2), while whenever $p' > 0$ it tends to be more symmetric and evenly distributed (Fig. 3). The size of the derivative of the probability function (i.e., the magnitude of $B$) instead determines where most of the mass is concentrated, and in particular whether the probability of low prevalence levels is higher or lower. However, the size of the derivative and its sign jointly contribute to determine such a feature of the invariant probability. When $p' < 0$ a high $B$ leads the distribution to be more concentrated toward the upper extreme of the support (Fig. 2), while when $p' > 0$ a high $B$ leads it to be more concentrated toward the lower extreme (Fig. 3).

We can conclude that the monotonicity (increasingness vs. decreasingness) and the steepness (low vs. high steepness) properties of the state-dependent probability function jointly contribute to determine a wide variety of possible outcomes. There may be situations in which it is fair to expect that the steady state outcome will be associated with positive but low levels of disease prevalence, and as the spread of the invariant distribution is particularly small in the stochastic steady state it is almost possible to deterministically determine the arising prevalence level ($p' < 0$ and $B$ small—see Fig. 2, top panels). Alternatively, it may happen that the spread of the invariant distribution is as large as its support such that in the stochastic steady state it is almost impossible to forecast the arising prevalence level ($p' < 0$ and $B$ large—see Fig. 2, bottom panels). But it may also happen that, despite the more limited spread, disease prevalence is evenly spread across the support such that we cannot really understand whether prevalence will tend to be characterized by low or high values ($p' > 0$, both with small and large $B$—see Fig. 3).

Our results surprisingly suggest that behavioral changes aiming at reducing individuals' exposure to the disease ($p' > 0$) may not always be that desirable in improving the long run health outcome, since people's behavioral response to changes in disease prevalence combined with the random diffusion of new disease strains may result in perpetual epidemic waves characterized by eventually high prevalence. But also the absence of behavioral changes to minimize disease exposure ($p' < 0$) may not be more desirable as in this case this may generate monotonic epidemic dynamics giving rise to high prevalence. Therefore, whenever the probability of epidemic shocks is state dependent it may be more important than ever to rely on public intervention to improve the long run health outcomes. We thus now investigate the role of public disease containment policies in shaping the invariant distribution of prevalence under state-dependent probabilities.

## 3 The economic model

We now introduce our economic framework by analyzing how a social planner decides the intensity of the policy measure to reduce the spread of a communicable disease, whose evolution is characterized as in the previous section, in order to minimize the social cost associated with the epidemic management program. As in epidemic periods the control of the spread of the communicable disease becomes the main priority for policymakers, we assume that the resources available to contain the epidemic are unconstrained, that is policymakers may always rely on international borrowing to finance their mitigation expenditure needs. For the sake of simplicity we do not model international borrowing, but we simply assume that the resources available for public health policy are exogenously given and large enough to meet policymakers' expenditure needs. The disease dynamics is characterized as in the previous section by the following equation: $I_{t+1} = \Omega z_t I_t + \theta z_t - X_t$, where the last term, $X_t \geq 0$, captures the effects of treatment measures which, by favoring recovery, reduces the number of infectives. The social cost is the discounted sum ($0 < \beta < 1$ is the discount factor) of the one-period losses associated with the epidemic management program. The one-period loss function depends on the level of disease incidence, $z_t I_t$, the potential infections associated with the diffusion of a new strain of the disease, $z_t S_t$ and the intensity of policy intervention, and is assumed to take the following additively separable quadratic form: $\ell(I_t, S_t, z_t) = \gamma_1 z_t^2 I_t^2 + \gamma_2 z_t^2 S_t^2 + X_t^2$, where $\gamma_1 > 0$ and $\gamma_2 > 0$ measure the relative weight of incidence and potential infection with respect to economic policy, respectively. Note that the potential infections due to a new disease strain depend on the share of susceptibles, since only susceptible individuals may be subject to infection (infectives are already exposed to the disease, thus the diffusion of a new strain may affect the economy only up to the extent that its population is susceptible). The random shock term $z_t$ directly affects the instantaneous losses since the diffusion of a new strain determines disease incidence and potential infections. By recalling that $S_t = 1 - I_t$ and denoting with $\mathbb{E}_0$ the expectation operator at time $t = 0$, the social planner's problem can be summarized by the following stochastic dynamic programming model:

$$V(I_0, z_0) = \min_{\{X_t\}} \mathbb{E}_0 \sum_{t=0}^{\infty} \beta^t \left[ \gamma_1 z_t^2 I_t^2 + \gamma_2 z_t^2 (1 - I_t)^2 + X_t^2 \right]$$

$$\text{s.t.} \begin{cases} I_{t+1} = \Omega z_t I_t + \theta z_t - X_t, \\ 0 \le I_t \le 1, \ X_t \ge 0 \ \forall t \ge 0, \\ 0 \le I_0 \le 1 \text{ and } z_0 \in \{r_1, r_2\} \text{ are given,} \end{cases} \tag{11}$$

where $\{z_t\}_{t=0}^{\infty}$ is the Bernoulli process (3) taking positive values $r_1, r_2$ such that $r_1 < r_2$ with *state-dependent probabilities* $p(I_t)$ and $1 - p(I_t)$ discussed in the previous section, and where the probability function is alternatively specified as in (4) or (5).

As by assumption $X_t \ge 0$ must hold for all $t \ge 0$, $I_{t+1} = \Omega z_t I_t + \theta z_t - X_t \le \Omega z_t I_t + \theta z_t \le 1$ holds for all $t \ge 0$, where the last inequality is a consequence of condition (8), $0 < r_1 < r_2 \le \frac{1}{\eta \tilde{\Omega}}$, discussed in Sect. 2. Moreover, the value $I_{t+1} = \Omega z_t I_t + \theta z_t - X_t = 0$ is always feasible for any $I_t$ value and shock realization $z_t$ because $X_t$ can be taken large enough. Hence, we can substitute $X_t = \Omega z_t I_t + \theta z_t - I_{t+1}$ from the dynamic constraint into the one-period objective function so that the reduced problem associated with (11) can be stated as follows:

$$V(I_0, z_0) = \min_{\{I_t\}} \mathbb{E}_0 \sum_{t=0}^{\infty} \beta^t \left[ \gamma_1 z_t^2 I_t^2 + \gamma_2 z_t^2 (1 - I_t)^2 + (\Omega z_t I_t + \theta z_t - I_{t+1})^2 \right]$$

$$\text{s.t.} \begin{cases} 0 \le I_{t+1} \le \Omega z_t I_t + \theta z_t, \ \forall t \ge 0, \\ 0 \le I_0 \le 1 \text{ and } z_0 \in \{r_1, r_2\} \text{ are given,} \end{cases} \tag{12}$$

where the constraint $0 \le I_t \le 1$ for all $t \ge 0$ is guaranteed by condition (8). Note that the probability $p(I_t)$ determines the occurrence of the random shock $z_t$ at the same time $t$ in which the actual number of infectives is $I_t$; hence, such a probability, through the realization of one of the two shocks $z_t \in \{r_1, r_2\}$, affects both the instantaneous losses in the objective function at time $t$ and the number of infectives in the next period $t + 1$ through the dynamic constraint. These properties ensure that disease prevalence follows a Markovian stochastic process, implying the validity of the dynamic programming principle which we will employ in the next section to determine the optimal policy, namely that the solution is time-consistent (Carpentier et al. 2012).

Because the $z_t$-sections of the graph $G = \{(I_t, I_{t+1}, z_t) : I_{t+1} \in \Gamma(I_t, z_t)\}$ of the optimal correspondence $\Gamma(k_t, z_t) = \{I_{t+1} : 0 \leq I_{t+1} \leq \Omega z_t I_t + \theta z_t\}$ are convex sets and the one-period objective function is quadratic, (12) is clearly a *convex problem* defined over the state space [0, 1].

## 4 The optimal policy and dynamics

In order to explicitly determine the optimal policy in our economic-epidemiological model, we need to solve in closed-form the Bellman equation associated with (12), which reads as follows:

$$V(I, z) = \min_{0 \leq y \leq \Omega z I + \theta z} \Big[ \gamma_1 z^2 I^2 + \gamma_2 z^2 (1 - I)^2 + (\Omega z I + \theta z - y)^2 + \beta \mathbb{E}_y V(y, z') \Big], \tag{13}$$

where $\mathbb{E}_y$ denotes the expectation operator that depends on the probabilities of both realizations of the random variable $z'$ occurring in the next period, themselves depending on the choice $y$, which corresponds to the number of infectives in the next period; that is, $\Pr(z' = r_1) = p(y) = \frac{1}{By^2+1}$ and $\Pr(z' = r_2) = 1 - p(y) = \frac{By^2}{By^2+1}$ if probabilities are taken according to (4), or $\Pr(z' = r_1) = p(y) = \frac{By^2}{By^2+1}$ and $\Pr(z' = r_2) = 1 - p(y) = \frac{1}{By^2+1}$ if probabilities are taken according to (5) (recall that, for given $y$, the random variable $z'$ is independent of past realizations, a property that guarantees the time-consistency of the solution, as $p(\cdot)$ depends only on the number of infectives $y$ planned by policymakers for the next period). Using these closed forms for $p(y)$ and $1 - p(y)$, the expectation $\mathbb{E}_y$ in the Bellman equation (13) can be directly evaluated and subsequently solved.

However, it turns out that, if we aim at keeping all the parameter values $\eta$, $\tilde{\Omega}$, $\theta$, $r_1$, $r_2$, $\beta$ and $\gamma_2$ fixed, the solution of the Bellman equation (13) is determined by two different values for the critical parameter $B$ characterizing the probabilities $p(y)$ and $1 - p(y)$ (as well as, incidentally, $\gamma_1$) for the decreasing probability case (4) and the increasing probability case (5). Therefore, we must consider separately the two cases in which the state-dependent probabilities have either the form in (4) or in (5).

### 4.1 The $p' < 0$ case

We start by analyzing the case in which the state-dependent probability function $p(y)$ is decreasing. The following proposition characterizes the closed-form solution for the Bellman equation along with the optimal policy (its proof is a special case of the proof of Theorem 2 in Sect. 6, reported in "Appendix C", in the case in which $N = 2$, and state-dependent probabilities are defined according to (27) with $a_1 = 0$, $b_1 = 1$, $a_2 = 1$ and $b_2 = 0$.).

**Proposition 1** *Let* $0 < \beta < 1$, $\tilde{\Omega} > 0$, $\eta > 0$, $0 < \theta < \eta\tilde{\Omega}$; *set* $\Omega = \eta\tilde{\Omega} - \theta$, *choose* $\gamma_2$ *such that* $0 < \gamma_2 < \theta\Omega$, *and assume that the shocks' values satisfy the feasibility*

*condition* (8), $0 < r_1 < r_2 \leq \frac{1}{\eta\tilde{\Omega}}$*.Assume that the state-dependent probabilities are defined as in* (4), $p(I) = \frac{1}{BI^2+1}$ *and* $1 - p(I) = \frac{BI^2}{BI^2+1}$, *where*

$$B = \frac{\Omega}{\beta\left(\theta\Omega - \gamma_2\right)\left(\Omega + \theta\right) r_2^2}. \tag{14}$$

*If, moreover, parameter* $\gamma_1$ *is given by*

$$\gamma_1 = \left[\frac{1}{\beta\left(\theta\Omega - \gamma_2\right) r_2^2} - \frac{\Omega + \theta}{\theta}\right]\gamma_2, \tag{15}$$

*then, the solution of the Bellman equation* (13) *is the function* $V(I, z) = Az^2\left(BI^2 + 1\right) + C$ *where B is given by* (14),

$$A = \frac{\Omega + \theta}{\Omega}\gamma_2 \quad \text{and} \quad C = \frac{\beta\left(\Omega + \theta\right) r_1^2}{\left(1 - \beta\right)\Omega}\gamma_2. \tag{16}$$

*The optimal policy for the number of infectives is affine in* $I_t^*$ *and has the following form:*

$$I_{t+1}^* = h\left(I_t^*, z_t\right) = \frac{\theta\Omega - \gamma_2}{\theta} z_t I_t^* + \frac{\theta\Omega - \gamma_2}{\Omega} z_t, \tag{17}$$

*while the corresponding optimal policy parameter is given by:*

$$X_t^* = \frac{\gamma_2}{\theta} z_t I_t^* + \frac{\gamma_2}{\Omega} z_t = \gamma_2\left(\frac{1}{\theta} I_t^* + \frac{1}{\Omega}\right) z_t. \tag{18}$$

The affine optimal policy in (17) can be rewritten in terms of the following IFSSDP:

$$I_{t+1} = \begin{cases} \dfrac{\theta\Omega - \gamma_2}{\theta} r_1 I_t + \dfrac{\theta\Omega - \gamma_2}{\theta} r_1 & \text{with probability } p\left(I_t\right) = \dfrac{1}{BI^2 + 1} \\ \dfrac{\theta\Omega - \gamma_2}{\theta} r_2 I_t + \dfrac{\theta\Omega - \gamma_2}{\theta} r_2 & \text{with probability } 1 - p\left(I_t\right) = \dfrac{BI^2}{BI^2 + 1}, \end{cases} \tag{19}$$

where the constant $B$ corresponds to the value in (14). There exists a unique stationary distribution $\mu$ for such an IFSSDP supported on the interval $\left[I_1^{st}, I_2^{st}\right] \subset [0, 1]$, where the endpoints are the steady states of the two affine maps in (19) respectively:

$$I_1^{st} = \frac{\left(\theta\Omega - \gamma_2\right)\theta r_1}{\Omega\left[\theta - \left(\theta\Omega - \gamma_2\right) r_1\right]} \quad \text{and} \quad I_2^{st} = \frac{\left(\theta\Omega - \gamma_2\right)\theta r_2}{\Omega\left[\theta - \left(\theta\Omega - \gamma_2\right) r_2\right]}. \tag{20}$$

It is straightforward to show that the steady states of the maps above are strictly lower than those in the absence of policy intervention—see (7). This suggests that containment policy is effective as it moves leftward the support of the invariant distribution, meaning that the steady state disease prevalence will tend to be characterized by lower values than what we would observe without any containment effort. Moreover, as both expressions in (20) are increasing in $\Omega = \eta\tilde{\Omega} - \theta$ (that is, increasing in $\eta$ and/or $\tilde{\Omega}$ for $\theta$ fixed) and decreasing in $\gamma_2$, the smaller $\Omega$ and/or the larger $\gamma_2$, the larger the leftward shift of the support. It is more difficult to isolate the effects of the parameter $\theta$ as, after substituting $\Omega$ with $\eta\tilde{\Omega} - \theta$, the expressions of $I_1^{st}$ and $I_N^{st}$ become more cumbersome: numerical examples show a non-monotonic pattern of both steady states as $\theta$ increases between 0 and $\eta\tilde{\Omega}$.

We now present a numerical example to clarify how optimal behavior by a social planner may affect the characteristics of the invariant distribution with respect to that approximated in Fig. 2c of Sect. 2 for the same epidemiological parameter values as in (9), and setting the remaining economic parameters as follows:

$$\beta = 0.96, \qquad \gamma_2 = 0.25, \tag{21}$$

which from expressions (14), (15) and (16) in Proposition 1 imply that

$$B = 3.571, \qquad \gamma_1 = 0.589, \qquad A = 0.375, \qquad C = 0.25.$$

Note that the value of the parameter $B$ is exactly the same we have used in Sect. 2 for the first case characterized by $p' < 0$, i.e., when $p(I_t)$ and $1 - p(I_t)$ are defined according to (4); this allows us to compare the steady state outcome arising in the same setting with and without containment policy.

According to (19) the optimal policy is represented by the following IFSSDP:

$$I_{t+1} = \begin{cases} 0.292 I_t + 0.146 & \text{with probability } p(I_t) = \dfrac{1}{3.571 I_t^2 + 1} \\[2ex] 0.583 I_t + 0.292 & \text{with probability } 1 - p(I_t) = \dfrac{3.571 I_t^2}{3.571 I_t^2 + 1}, \end{cases} \tag{22}$$

which has $\left[I_1^{st}, I_2^{st}\right] = [0.206, 0.7]$ as trapping interval. As the fixed point of the lower map, $I_1^{st} = 0.206$, is bounded away from 0, clearly both $p(I_t)$ and $1 - p(I_t)$ are such that $0 < p(I_t) < 1$ and $0 < 1 - p(I_t) < 1$ for all $I \in [0.206, 0.7]$. Figure 4 shows the initial uniform density $\mu_0(I) \equiv \frac{1}{I_2^{st} - I_1^{st}}$ and the 1st and 6th iterations of our Maple algorithm for the IFSSDP (22), where the last plot can be considered as a good approximation of the invariant measure in this case. Note that, as $0.292 I_2^{st} + 0.146 = 0.35 < 0.412 = 0.583 I_1^{st} + 0.292$, the images of the two affine maps in (22) do not overlap, so that the invariant measure is singular as it is supported on a Cantor-like set.

By comparing Fig. 2 (top panels) with Fig. 4, exactly as we have discussed before, we can observe that the support of the invariant probability measure in the latter is characterized by lower extremes than those in the absence of containment policy of the former. Moreover, we can see that the effects of the optimal mitigation policy consist of concentrating the value of disease prevalence more closely toward to the lower

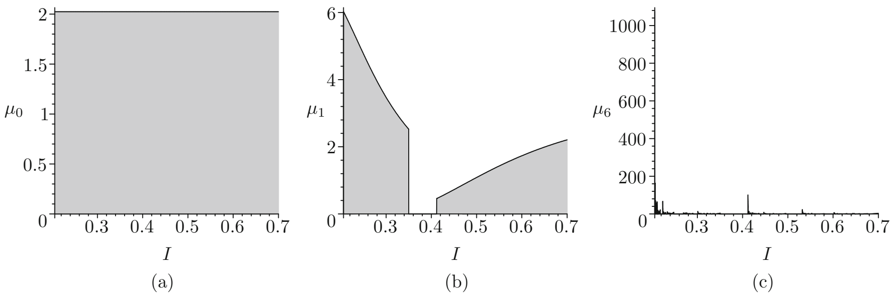

**Fig. 4** **a** initial uniform density over [0.206, 0.7], **b** 1st and **c** 6th iterations of our Algorithm to approximate the Markov operator (46) associated to the IFSSDP (22)

extreme of the support, $I_1^{st} = 0.206$, as it becomes apparent by comparing directly Figs. 2c and 4c; in fact, the latter plot exhibits a much higher spike close to $I_1^{st}$ than that in the former figure. Thus, containment policy not only reduces on average the possible steady state values of disease prevalence, but it also increases the likelihood that prevalence will be associated with its possible lowest values.

It will be shown in the next Proposition 2 that, in order to keep all parameters' values—except for $\gamma_1$—the same as in (9) and (21), the value of parameter $B$ in the expression of the value function $V(I, z) = Az^2\left(BI^2 + 1\right) + C$ turns out to be different than the expression in (14) when the probability $p(I)$ is increasing. Therefore, Proposition 1 cannot be applied for the $p' > 0$ case when $B = 3.571$. That is, we have no comparison between the optimal dynamics determined by containment policies and the dynamics described in Fig. 3 (top panels). To compare the dynamics with and without containment policies when $p' > 0$ we must resort to the result in the next Proposition 2 specifically designed for the increasing $p(I)$, corresponding to the value $B = 14.286$.

### 4.2 The $p' > 0$ case

We now analyze the case in the which the state-dependent probability function $p(y)$ is increasing. The closed-form solution for the Bellman equation and the optimal policy are determined in the following proposition (its proof is a special case of the proof of Theorem 2 in Sect. 6, reported in "Appendix C", in the case in which $N = 2$, and state-dependent probabilities are defined according to (27) with $a_1 = 1$, $b_1 = 0$, $a_2 = 0$ and $b_2 = 1$).

**Proposition 2** *Let* $0 < \beta < 1$, $\tilde{\Omega} > 0$, $\eta > 0$, $0 < \theta < \eta\tilde{\Omega}$; *set* $\Omega = \eta\tilde{\Omega} - \theta$, *choose* $\gamma_2$ *such that* $0 < \gamma_2 < \theta\Omega$, *and assume that the shocks' values satisfy the feasibility condition* (8), $0 < r_1 < r_2 \leq \frac{1}{\eta\tilde{\Omega}}$. *Assume that the state-dependent probabilities are defined as in* (5), $p(I) = \frac{BI^2}{BI^2+1}$ *and* $1 - p(I) = \frac{1}{BI^2+1}$, *where*

$$B = \frac{\Omega}{\beta(\theta\Omega - \gamma_2)(\Omega + \theta)r_1^2}. \tag{23}$$

*If, moreover, parameter* $\gamma_1$ *is given by*

$$\gamma_1 = \left[ \frac{1}{\beta\left(\theta\Omega - \gamma_2\right) r_1^2} - \frac{\Omega + \theta}{\theta} \right] \gamma_2, \tag{24}$$

*then, the solution of the Bellman equation* (13) *is the function* $V(I, z) = Az^2\left(BI^2 + 1\right) + C$ *where* $B$ *is given by* (23),

$$A = \frac{\Omega + \theta}{\Omega} \gamma_2 \quad \text{and} \quad C = \frac{\beta\left(\Omega + \theta\right) r_2^2}{(1 - \beta)\,\Omega} \gamma_2. \tag{25}$$

*The optimal policies for the number of infectives* $I_t^*$ *and the corresponding parameter* $X_t^*$ *are the same as in expressions* (17) *and* (18) *in Proposition* 1.

Because the optimal policy is the same as in (17) of Proposition 1 in the previous subsection, the IFSSDP describing the optimal dynamics of the number of infectives is the same as in (19), only with state-dependent probabilities defined by (5) instead of (4), themselves characterized by different values of the parameter $B$. Note the apparently slight—but substantial in terms of numerical values—difference in the expressions of parameter $B$ as in (14) and in (23), and of parameter $\gamma_1$ as in (15) and in (24). The difference is entirely driven by the shock value in the denominators, which is $r_2^2$ in Proposition 1 while it is $r_1^2$ in Proposition 2. Similarly, also parameter $C$—which does not affect the optimal policy—has different expressions in (16) and in (25 ). Also in this case of an increasing state-dependent probability function $p(y)$ it is possible to prove the existence of a unique stationary distribution $\mu$ for such an IFSSDP supported on the interval $\left[I_1^{st}, I_2^{st}\right] \subset [0, 1]$, where the endpoints are the same steady states of the two affine maps in (19) given by (20). Hence, the same comments apply: containment policy is effective as it results in a leftward shift of the support of the invariant distribution, meaning that steady state disease prevalence will be characterized on average by lower values than in the absence of policy intervention.

We continue to illustrate numerically how optimal behavior by a social planner may affect the characteristics of the invariant distribution with respect to that approximated in Fig. 3f of Sect. 2 for the same epidemiological parameter' values as in (9) and (21). Expressions (23), (24) and (25) in Proposition 2 imply that:

$$B = 14.286, \qquad \gamma_1 = 4.607, \qquad A = 0.375, \qquad C = 1.$$

Note that also in this case the value of the parameter $B$ is exactly the same of that used in Sect. 2 for the second case characterized by $p' > 0$; this allows us to compare the steady state outcome arising in the same setting with and without mitigation policy. Now the constant $B$ has a much larger value than in the $p' < 0$ case; this

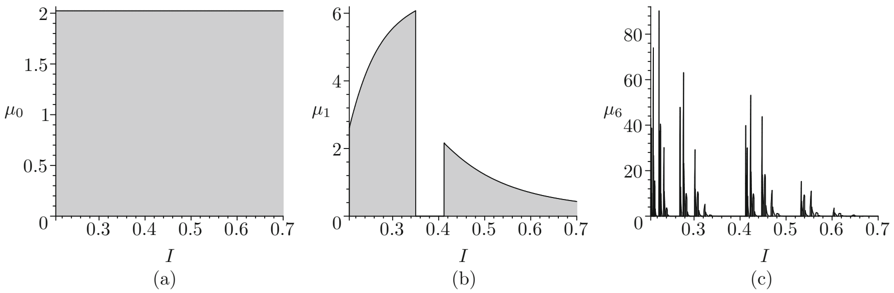

**Fig. 5** **a** initial uniform density over [0.206, 0.7], **b** 1st and **c** 6th iterations of our Algorithm to approximate the Markov operator (46) associated to the IFSSDP (26)

feature translates into much steeper state-dependent probabilities. According to (17) the optimal policy is represented by the following IFSSDP:

$$I_{t+1} = \begin{cases} 0.292 I_t + 0.146 & \text{with probability } p\left(I_t\right) = \dfrac{14.286 I_t^2}{14.286 I_t^2 + 1} \\ 0.583 I_t + 0.292 & \text{with probability } 1 - p\left(I_t\right) = \dfrac{1}{14.286 I_t^2 + 1}, \end{cases} \tag{26}$$

which has the same interval, $\left[I_1^{st}, I_2^{st}\right] = [0.206, 0.7]$, as the IFSSDP (22) as trapping interval. Figure 5, as usual, shows the initial uniform density $\mu_0\left(I\right) \equiv \frac{1}{I_2^{st} - I_1^{st}}$ and the 1st and 6th iterations of our Maple algorithm for the IFSSDP (26). As the affine maps are the same as in (22), again their images do not overlap and the invariant measure is singular as it is supported on a Cantor-like set.

By comparing Fig. 3 (bottom panels) with Fig. 5, exactly as in the $p' < 0$ case, we can observe that the support of the distribution is characterized by lower extremes than in the absence of containment policy, and that the optimal containment policy results in concentrating the value of disease prevalence more closely toward to the lower extreme of the support, as it becomes apparent by comparing directly Figs. 3f and 5c; in fact, the latter plot exhibits higher spikes close to $I_1^{st}$ than those in the former figure, although in a less pronounced fashion than in our earlier comparison between Figs. 2c and 4c. Thus, once more, containment policy not only reduces on average the possible steady state values of disease prevalence, but it also increases the likelihood that prevalence will be associated with its possible lowest values.

A similar explanation as that given at the end of Sect. 4.1 applies to the $p' > 0$ case: a comparison between the dynamics under optimal containment policies when $B = 14.286$ and the dynamics without policies described by Fig. 3 (bottom panels) for the $p' < 0$ case is not available. Again, in order to keep all parameters' values—except for $\gamma_1$—the same as in (9) and (21), the value $B = 14.286$ needs to be considered in the $p' > 0$ case according to Proposition 2. The value $B = 3.571$ can be applied for the same parameters' values only when $p' < 0$, according to Proposition 1.

## 5 Singularity versus absolute continuity

One important question, especially from a policy perspective, regarding the nature of the invariant distribution $\mu$ is related to its properties in terms of absolutely continuity or singularity. Specifically, if it is absolutely continuous then it will be represented by a density and so it could be estimated in terms of a few parameters, while if it is singular then there will be no convenient way to represent it and we will have to list the value of the function for every point in its domain. Clearly, absolutely continuous measures are easier to work with and more well-behaved than singular measures as they allow for a more precise forecasting of future dynamics, and so it is valuable to know conditions under which the invariant measure may be absolutely continuous. There is also a long history of works in this area and it is still a very active area of theoretical research. The strongest results are when the contraction factors are all the same (so-called *equi-contractive IFS*). Our situation with unequal scaling factors is more delicate and so we are only able to give an incomplete characterization. In the constant probability case, recent work in Saglietti et al. (2018) shows that for each fixed choice of probability the invariant measure is absolutely continuous for almost every $(\alpha, \beta)$ in the so-called "super-critical region". Our situation with state-dependent probabilities is quite a bit more intricate so our result is less comprehensive. The description of the region $\Theta$ is complicated and can be found in Ngai and Wang (2005). We can prove the following result (whose proof is presented in "Appendix B").

**Theorem 1** *Take the two-map IFS on* $\mathcal{R}$ *given by* $\{\alpha x + \tau_1, \beta x + \tau_2\}$, *with* $\alpha, \beta \in [0, 1)$ *along with the two probability functions* $p_1(x) = p(x)$ *and* $p_2(x) = 1 - p(x)$. *Assume that* $\delta < p(x) < 1 - \delta$ *for all* $x$ *and some* $\delta > 0$ *and also that* $p$ *is Hölder continuous. Let* $\mu_{\alpha,\beta}$ *be the invariant measure of this state-dependent IFS.*

1. *If* $0 \leq \alpha + \beta < 1$ *then* $\mu_{\alpha,\beta}$ *is singular with respect to Lebesgue measure.*
2. *If* $\alpha + \beta = 1$ *then* $\mu_{\alpha,\beta}$ *is either singular with respect to Lebesgue measure or is equal to the (normalized) Lebesgue measure on the closed interval with endpoints* $\frac{\tau_1}{1-\alpha}$ *and* $\frac{\tau_2}{1-\beta}$ *and* $p(x) = \alpha$.
3. *For each* $\alpha + \beta > 1$, *let* $h_{\alpha,\beta}$ *be defined by*

$$h_{\alpha,\beta} = -\int \{p(x)\ln[p(x)] + [1 - p(x)]\ln[1 - p(x)]\} d\mu_{\alpha,\beta}(x)$$

*and*

$$\chi_{\alpha,\beta} = -\log(\beta) + [\log(\beta) - \log(\alpha)] \int p(x)\, d\mu_{\alpha,\beta}(x).$$

*Then* $\mu_{\alpha,\beta}$ *is singular for every* $\alpha, \beta$ *with* $h_{\alpha,\beta} < \chi_{\alpha,\beta}$.

*Furthermore, there is an open subset* $\Theta \subset \{(\alpha, \beta) \in (0, 1)^2 : \alpha + \beta > 1\}$ *so that* $\mu_{\alpha,\beta}$ *is absolutely continuous with respect to Lebesgue measure for Lebesgue almost every* $(\alpha, \beta) \in \Theta$ *such that* $h_{\alpha,\beta} > \chi_{\alpha,\beta}$.

Theorem 1 states that the singularity vs. absolute continuity properties of the invariant measure depend ultimately on the contraction factors, which in our IFSSDPs is given by $\frac{\theta\Omega-\gamma_2}{\theta}=\frac{\theta\left(\eta\tilde{\Omega}-\theta\right)-\gamma_2}{\theta}$, and thus it depends on the relative magnitude of the net infectivity rate, $\tilde{\Omega}$, the extent to which the shock realization affects the net infectivity rate and susceptibles, $\eta$ and $\theta$, and the weight attached to potential infections in the objective function, $\gamma_2$. While it is straightforward to check whether one of the first two cases of the theorem applies, the third case is quite a bit more delicate and deserves some further clarification. In fact, the condition $h_{\alpha,\beta} > \chi_{\alpha,\beta}$ is generally difficult to check since it involves integrals with respect to $\mu$. Moreover, unfortunately for any specific choice of parameters it is not a simple task to determine if $\mu$ is absolutely continuous even if this condition holds. All we would know is that $\mu$ is absolutely continuous for almost all choices of the parameters in some open subset. Even in the case of equal contraction factors it would be difficult to know if a specific choice of parameters results in an absolutely continuous invariant measure. We do know, however, that the invariant distribution is a continuous function of the parameters with respect to the Monge-Kantorovich metric.

Returning to our epidemiological framework, all the IFSSDPs that we have analyzed, both in the case of presence and absence of optimal disease containment policies, fit into the scheme of Theorem 1 as there are two random shocks, the IFS maps are both one-dimensional, and affine and the probabilities are smooth functions. The IFSSDPs with optimal policy given in (22) and (26) are both in the first case of the theorem (where $\alpha + \beta < 1$) and thus their invariant measures are singular, and this is true no matter the form of the probability function $p(x)$. The IFSSDP without mitigation policy given in (10) is in the second case where $\alpha + \beta = 1$. However, since the probability functions are not constant (and equal in value to the corresponding contraction ratios), the invariant measure is singular also in this case. While none of our specific parametrizations have resulted in a IFSSDP fitting the third case where $1 < \alpha + \beta < 2$, this could be true in principle for any of our IFSSDPs, both without optimal policy—given in (6)—and with optimal policy—given in (19) with different probability characterizations (different values for parameter $B$) for either scenario, $p' < 0$ or $p' > 0$, respectively.

## 6 Extension

In order to present the implications of state-dependent probabilities in our economic-epidemiological framework in the most intuitive way, thus far we have assumed that random shocks are associated with the realization of only two alternative outcomes. This has allowed us to understand how the characteristics (in terms of monotonicity and steepness properties) of the probability function associated with the lowest shock value affects epidemic dynamics and the optimal containment policy. We now relax this assumption by assuming that random shocks can take on one of a finite number $N$ of values, $z_t \in \{r_1, \dots, r_N\}$, such that $0 < r_1 < \dots < r_N$, and thus in this context with multiple state-dependent probabilities we can no longer isolate the separate role played by each state-dependent probability function, apart from some particular cases (as for example the one that we shall discuss later).

We consider the following hyperbolic form for the probabilities $p_i(\cdot)$, defined for $I \in [0, 1]$:

$$p_i(I) = \frac{Ba_i I^2 + b_i}{BI^2 + 1}, \qquad i = 1, \ldots, N, \tag{27}$$

where $B > 0$, $a_i \geq 0$, and $b_i \geq 0$ are parameters satisfying $\sum_{i=1}^{N} a_i = \sum_{i=1}^{N} b_i = 1$. Note that the above functional form is a generalization of the functional forms we have previously introduced in our analysis, as it contains both our decreasing and increasing probability functions as special cases. The above hyperbolic form (27) defines $N$ (Lipschitz) continuous state-dependent probability functions satisfying $0 \leq p_i(I) \leq 1$ for all $0 \leq I \leq 1$ and $i = 1, \ldots, N$. As we have done in our previous analysis, in the following we shall choose parameter values such that the dynamics of $I_t$ will always remain trapped in a proper sub-interval of [0, 1], so that the values of $p_i(I)$ will always be bounded away from 0 and 1 (consistent with Theorem 3 and Corollary 1 in "Appendix A" to ensure the existence and uniqueness of the invariant measure).

The dynamics in (2) can be written in terms of the following IFSSDP:

$$I_{t+1} = \Omega r_i I_t + \theta r_i \qquad \text{with probability } p_i(I_t), \qquad \text{for } i = 1, \ldots, N \tag{28}$$

The same arguments presented earlier in the $N = 2$ case apply, and thus it is possible to prove the existence of a unique stationary distribution $\mu$ for such an IFSSDP supported on the interval $\left[I_1^{st}, I_N^{st}\right] \subset [0, 1]$, where the endpoints are the steady states of the lowest and highest affine maps in (28) respectively:

$$I_1^{st} = \frac{\theta r_1}{1 - \Omega r_1} \qquad \text{and} \qquad I_N^{st} = \frac{\theta r_N}{1 - \Omega r_N}. \tag{29}$$

Similar to what we have discussed in our baseline model, in order to keep the dynamics of $I_t$ defined by IFSSDP (28) inside the interval [0, 1], the steady states of the above maps must satisfy the following parameter condition, which generalizes condition (8):

$$0 < r_1 < \cdots < r_N \leq \frac{1}{\eta \tilde{\Omega}}. \tag{30}$$

The reduced optimization problem presented below is the same as in (12), only with $N$ possible realizations for the shock $z_t$:

$$V(I_0, z_0) = \min_{\{I_t\}} \mathbb{E}_0 \sum_{t=0}^{\infty} \beta^t \left[\gamma_1 z_t^2 I_t^2 + \gamma_2 z_t^2 (1 - I_t)^2 + (\Omega z_t I_t + \theta z_t - I_{t+1})^2\right]$$
$$\text{s.t.} \begin{cases} 0 \leq I_{t+1} \leq \Omega z_t I_t + \theta z_t, \ \forall t \geq 0, \\ 0 \leq I_0 \leq 1 \text{ and } z_0 \in \{r_1, \ldots, r_N\} \ \text{are given.} \end{cases} \tag{31}$$

Again, the $z_t$-sections of the graph $G = \{(I_t, I_{t+1}, z_t) : I_{t+1} \in \Gamma(I_t, z_t)\}$ of the optimal correspondence $\Gamma(k_t, z_t) = \{I_{t+1} : 0 \leq I_{t+1} \leq \Omega z_t I_t + \theta z_t\}$ are convex sets, so that, as the one-period objective function is quadratic, (12) is clearly a *convex problem* defined over the state space [0, 1].

Its associated Bellman equation is the same as in (13), with the only difference that now the expectation operator $\mathbb{E}_y$ is defined by the probabilities in (27), $\Pr\left(z' = r_i\right) = p_i(y) = \frac{Ba_i y^2 + b_i}{By^2 + 1}$ for $i = 1, \ldots, N$, so that $\mathbb{E}_y$ can be directly evaluated and the Bellman equation can be rewritten in the following form:

$$\begin{aligned} V(I, z) &= \min_{0 \leq y \leq \Omega z I + \theta z} \Bigg[\gamma_1 z^2 I^2 + \gamma_2 z^2 (1 - I)^2 + (\Omega z I + \theta z - y)^2 \\ &\quad + \beta \sum_{i=1}^{N} p_i(y) V(y, r_i)\Bigg] \\ &= \min_{0 \leq y \leq \Omega z I + \theta z} \Bigg[\gamma_1 z^2 I^2 + \gamma_2 z^2 (1 - I)^2 + (\Omega z I + \theta z - y)^2 \\ &\quad + \beta \sum_{i=1}^{N} \left(\frac{Ba_i y^2 + b_i}{By^2 + 1}\right) V(y, r_i)\Bigg]. \end{aligned}$$

In order to search for a closed-form solution of our optimization problem, we guess the following form for the value function in the Bellman equation:

$$V(I, z) = Az^2 \left(BI^2 + 1\right) + C,$$

where $A$, $B$ and $C$ are constants to be determined; specifically, $B$ is the same constant in the denominator of the state-dependent probabilities $p_i(I) = \frac{Ba_i I^2 + b_i}{BI^2 + 1}$. For such a quadratic guess the Bellman equation becomes:

$$\begin{aligned} V(I, z) &= Az^2 \left(BI^2 + 1\right) + C = \min_{0 \leq y \leq \Omega z I + \theta z} \Bigg\{\gamma_1 z^2 I^2 \\ &\quad + \gamma_2 z^2 (1 - I)^2 + (\Omega z I + \theta z - y)^2 \\ &\quad + \beta \sum_{i=1}^{N} \left(\frac{Ba_i y^2 + b_i}{By^2 + 1}\right) \left[Ar_i^2 \left(By^2 + 1\right) + C\right]\Bigg\} \\ &= \min_{0 \leq y \leq \Omega z I + \theta z} \Bigg[\gamma_1 z^2 I^2 + \gamma_2 z^2 (1 - I)^2 + (\Omega z I + \theta z - y)^2 \\ &\quad + \beta A \left(By^2 \sum_{i=1}^{N} a_i r_i^2 + \sum_{i=1}^{N} b_i r_i^2\right) + \beta C\Bigg]. \end{aligned} \tag{32}$$

The following result characterizes the closed-form solution for the Bellman equation (32) under some conditions on the model's parameters.

**Theorem 2** *Let* $0 < \beta < 1$, $\tilde{\Omega} > 0$, $\eta > 0$, $0 < \theta < \eta\tilde{\Omega}$; *set* $\Omega = \eta\tilde{\Omega} - \theta$, *choose* $\gamma_2$ *such that* $0 < \gamma_2 < \theta\Omega$, *and assume that the N shocks' values satisfy the feasibility condition* (30), $0 < r_1 < \cdots < r_N \leq \frac{1}{\eta\tilde{\Omega}}$. *Assume that the state-dependent probabilities are defined as in* (27), $p_i(I) = \frac{Ba_i I^2 + b_i}{BI^2+1}$, *for* $i = 1, \ldots, N$, *with* $a_i \geq 0$ *and* $b_i \geq 0$ *satisfying* $\sum_{i=1}^{N} a_i = \sum_{i=1}^{N} b_i = 1$, *where:*

$$B = \frac{\Omega}{\beta(\theta\Omega - \gamma_2)(\Omega + \theta)\sum_{i=1}^{N} a_i r_i^2}. \tag{33}$$

*If, moreover, parameter* $\gamma_1$ *is given by:*

$$\gamma_1 = \left[\frac{1}{\beta(\theta\Omega - \gamma_2)\sum_{i=1}^{N} a_i r_i^2} - \frac{\Omega + \theta}{\theta}\right]\gamma_2, \tag{34}$$

*then, the solution of the Bellman equation* (32) *is the function* $V(I, z) = Az^2\left(BI^2 + 1\right) + C$ *where B is defined in* (33) *and:*

$$A = \frac{\Omega + \theta}{\Omega}\gamma_2, \tag{35}$$

$$C = \frac{\beta(\Omega + \theta)\sum_{i=1}^{N} b_i r_i^2}{(1 - \beta)\Omega}\gamma_2. \tag{36}$$

*The optimal policy for the number of infectives is affine in* $I_t^*$ *and has the following form:*

$$I_{t+1}^* = h\left(I_t^*, z_t\right) = \frac{\theta\Omega - \gamma_2}{\theta} z_t I_t^* + \frac{\theta\Omega - \gamma_2}{\Omega} z_t, \tag{37}$$

*while the corresponding optimal policy parameter is given by:*

$$X_t^* = \frac{\gamma_2}{\theta} z_t I_t^* + \frac{\gamma_2}{\Omega} z_t = \gamma_2\left(\frac{1}{\theta} I_t^* + \frac{1}{\Omega}\right) z_t. \tag{38}$$

The proof of Theorem 2 is presented in "Appendix C", where it is shown that the expression of $\gamma_1$ in (34) is strictly positive. Clearly, as the constants $A$ and $B$ in (35) and (33) are strictly positive, the RHS of the Bellman equation (32) is strictly convex in $y$, so that the solution characterized in Theorem 2, including the optimal policy (37), is unique. As $0 < \gamma_2 < \theta\Omega$, clearly the optimal policy in (37) satisfies $0 < I_{t+1}^* < \Omega z_t I_t^* + \theta z_t \leq 1$ for all $t \geq 0$; similarly, the optimal policy parameter in (38) satisfies $0 < X_{t+1}^* < \Omega z_t I_t^* + \theta z_t \leq 1$ for all $t \geq 0$.

The affine optimal policy in (37) can be rewritten in terms of the following IFSSDP:

$$I_{t+1} = \frac{\theta\Omega - \gamma_2}{\theta} r_i I_t + \frac{\theta\Omega - \gamma_2}{\theta\Omega} r_i \quad \text{with probability } p\left(I_t\right) = \frac{B a_i I_t^2 + b_i}{B I_t^2 + 1}, \quad \text{for } i = 1, \ldots, N \tag{39}$$

where the constant $B$ corresponds to the value in (33) and $a_i$, $b_i$ are non-negative and satisfy $\sum_{i=1}^{N} a_i = \sum_{i=1}^{N} b_i = 1$. Again, also with containment policy, it is possible to prove the existence of a unique stationary distribution $\mu$ for such an IFSSDP supported on the interval $\left[I_1^{st}, I_N^{st}\right] \subset [0, 1]$, where the endpoints are the steady states of the lowest and highest affine maps in (39) respectively:

$$I_1^{st} = \frac{(\theta\Omega - \gamma_2)\,\theta r_1}{\Omega\left[\theta - (\theta\Omega - \gamma_2)\, r_1\right]} \quad \text{and} \quad I_N^{st} = \frac{(\theta\Omega - \gamma_2)\,\theta r_N}{\Omega\left[\theta - (\theta\Omega - \gamma_2)\, r_N\right]}. \tag{40}$$

As $I_1^{st}$ and $I_N^{st}$ in (40) have the same expressions as in (20), the same comments as in Sect. 4 apply: containment policy determines a leftward shift of the support of the invariant distribution, meaning that steady state disease prevalence will be characterized on average by lower values than in the absence of policy intervention. Moreover, the smaller $\Omega$ and/or the larger $\gamma_2$, the larger the leftward shift of the support. Exactly as before, we cannot characterize explicitly the implications of the state-dependency of the probability functions on the steady state distribution of disease prevalence, thus we need to proceed via numerical analysis. With multiple possible realizations of the shock value it is generally not possible to isolate the separate role played by each state-dependent probability function in determining the characteristics of the invariant distribution. Therefore, in our following discussion we will focus on a specific setup which allows us to obtain results comparable to those we have presented in the previous sections (i.e., the examples in Sects. 4.1 and 4.2), assessing the robustness of our previous conclusions.

Specifically, we now apply Theorem 2 to an IFSSDP characterized by three exogenous shocks (i.e., with three maps) and numerically simulate the 6th iteration of the Markov operator (46) in "Appendix A", showing that the main qualitative traits of the invariant measure in Figs. 2c, 3f, 4c and 5c are essentially preserved also in such a richer scenario in which the realization of random shocks may give rise to three alternative outcomes. To do so we keep the higher map as in (6) and (19) while we split the lower map in both IFSSDP (6) and (19) into two new maps, distributing the weights on each state-dependent probability defined as in (27) as follows: the higher map is characterized by the same probability as in our baseline model while the two lowest maps share similar probabilities as the one assumed in our baseline model for the unique lower map, with only a small fraction of it (equal to 0.1) associated with the third and lowest map and the remaining fraction (equal to 0.9) with the second and intermediate map.

We rely as much as possible on parameter values employed in our previous simulations, deviating from our previous parametrization only to satisfy the restriction imposed by Theorem 2. Hence, with $N = 3$ we keep $\eta = 3$, $\tilde{\Omega} = \theta = 1$, so that $\Omega = \eta\tilde{\Omega} - \theta = 2$, and set $r_3 = \frac{1}{\eta\tilde{\Omega}} = \frac{1}{3} \simeq 0.333$ (i.e., the same value as for the former $r_2$), so that condition (30) holds with equality. For the *almost* no-overlap property to hold for the images of the 3 maps defined according to (28) when there is no containment policy, we set $r_1 = \frac{2-\sqrt{3}}{6} \simeq 0.045$ and $r_2 = \frac{\sqrt{3}-1}{6} \simeq 0.122$; thus, according to (29), without containment policies now the support of the invariant measure is the interval $\left[I_1^{st}, I_3^{st}\right] = [0.049, 1]$, which is larger (to the left) than that in Sect. 2 due to the presence of a third lowest map in the IFSSDP (28), but still bounded away from 0. Therefore, under such a parameterization the IFSSDP (28) reads as follows:

$$I_{t+1} = \begin{cases} 0.089 I_t + 0.045 & \text{with probability } p_1(I_t) \\ 0.244 I_t + 0.122 & \text{with probability } p_2(I_t) \\ 0.667 I_t + 0.333 & \text{with probability } p_3(I_t), \end{cases} \tag{41}$$

with $\left[I_1^{st}, I_3^{st}\right] = [0.049, 1]$ as trapping interval. By keeping the parameter's value $\gamma_2 = 0.25$, the IFSSDP (39) defining the dynamic under the optimal containment policy turns out to be:

$$I_{t+1} = \begin{cases} 0.078 I_t + 0.039 & \text{with probability } p_1(I_t) \\ 0.214 I_t + 0.107 & \text{with probability } p_2(I_t) \\ 0.583 I_t + 0.292 & \text{with probability } p_3(I_t), \end{cases} \tag{42}$$

having $\left[I_1^{st}, I_3^{st}\right] = [0.042, 0.7]$ as trapping interval, where $I_1^{st}$ and $I_3^{st}$ are defined according to (40). Again, the attractor is larger (to the left) than that in Sect. 4 due to the presence of a third lowest map in the IFSSDP (42), but still bounded away from 0. As occurred in Sect. 4, it can be easily verified that the images of the three affine maps in (42) do not overlap, which implies that the invariant measure is singular, as it is supported on a Cantor-like set.

In order to extend the case with decreasing and increasing probabilities discussed in Sects. 4.1 and 4.2 to the IFSSDP (41) and (42), our approach to distribute the probability of the lowest map in our $N = 2$ case between the intermediate and lowest maps in our $N = 3$ framework yields, in the first example in which both probabilities for $w_1$ and $w_2$ are *decreasing*, the following probability functions: $p_1(I_t) = \frac{0.1}{BI_t^2+1}$, $p_2(I_t) = \frac{0.9}{BI_t^2+1}$ and $p_3(I) = \frac{BI^2}{BI^2+1}$. This implies that the three state-dependent probabilities as in (27) are characterized by coefficients $a_1 = 0$, $b_1 = 0.1$, $a_2 = 0$, $b_2 = 0.9$, and $a_3 = 1$, $b_3 = 0$. By maintaining also the parameter's value $\beta = 0.96$, according to (33 )–(36) of Theorem 2 such values for $a_i$ and $b_i$, in turn, yield:

$$B = 3.571, \qquad \gamma_1 = 0.589, \qquad A = 0.375, \qquad C = 0.122,$$

which are the same parameters' values as in Sect. 4.1 except for parameter $C$, while:

$$p_1(I_t) = \frac{0.1}{3.571 I_t^2 + 1}, \qquad p_2(I_t) = \frac{0.9}{3.571 I_t^2 + 1}, \qquad p_2(I_t) = \frac{3.571 I_t^2}{3.571 I_t^2 + 1}. \tag{43}$$

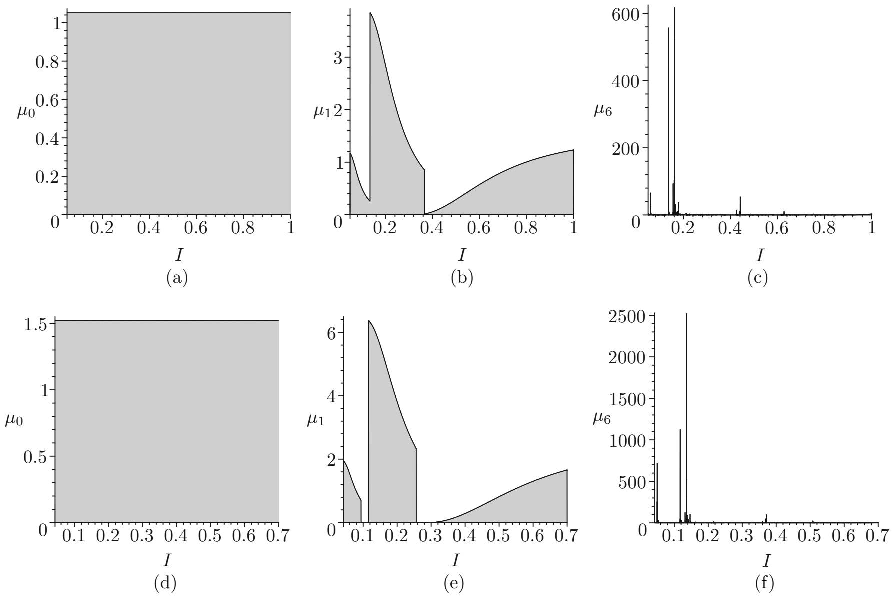

**Fig. 6** Initial uniform density (left), 1st (mid) and 6th (right) iterations of our Algorithm to approximate the Markov operator (46) associated to the IFSSDP (41) over [0.049, 1] (top) or the IFSSDP (42) over [0.042, 0.7] (bottom) when probabilities are given by (43)

Conversely, in the second example in which both probabilities for $w_1$ and $w_2$ are *increasing*, we set $p_1(I_t) = \frac{0.1BI^2}{BI_t^2+1}$, $p_2(I_t) = \frac{0.9BI^2}{BI_t^2+1}$ and $p_3(I) = \frac{1}{BI^2+1}$, such that the three state-dependent probabilities as in (27) are characterized by coefficients $a_1 = 0.1$, $b_1 = 0$, $a_2 = 0.9$, $b_2 = 0$, and $a_3 = 0$, $b_3 = 1$. According to (33)–(36) of Theorem 2 such values for $a_i$ and $b_i$, in turn, yield:

$$B = 29.185, \qquad \gamma_1 = 10.194, \qquad A = 0.375, \qquad C = 1.$$

Note that now $B$ and $\gamma_1$ have values quite higher than those obtained in Sect. 4.2, while $A$ and $C$ are the same,[3] In this case:

$$p_1(I_t) = \frac{2.919 I_t^2}{29.185 I_t^2 + 1}, \qquad p_2(I_t) = \frac{26.267 I_t^2}{29.185 I_t^2 + 1}, \qquad p_2(I_t) = \frac{1}{29.185 I_t^2 + 1}. \tag{44}$$

The results of our numerical analysis are shown in Figs. 6 and 7. Figure 6 focuses on our first example in which the probabilities of the first and second maps are both *decreasing*, that is $B = 3.571$ and probabilities $p_i(I)$s defined according to (43). Figure 7 focuses instead on our second example in which the probabilities of the first

[3] It is possible to show that even considering the higher value $B = 29.185$, our previous conclusions in the case of two shock values (i.e. $N = 2$) still apply. Specifically, from a qualitative point of view, the invariant distributions will look exactly like those illustrated in the previous sections.

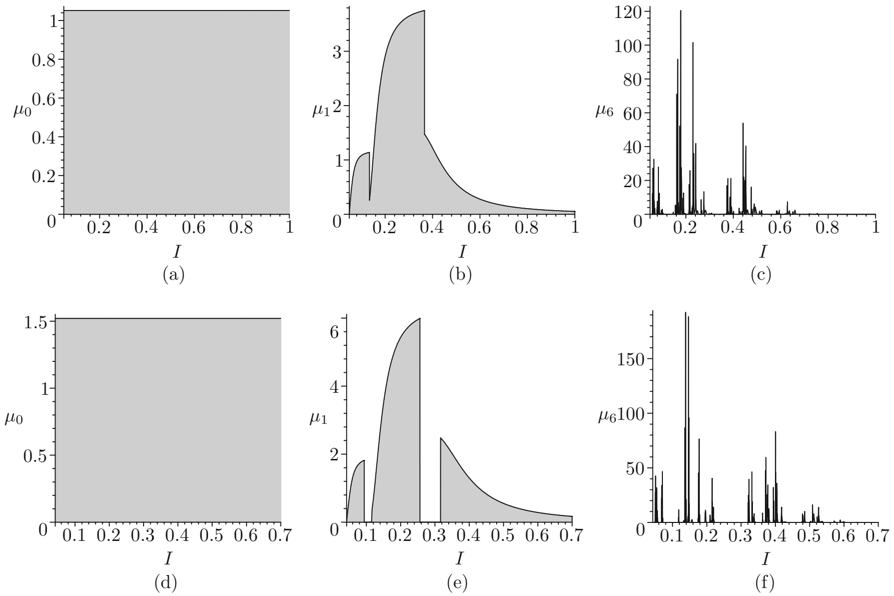

**Fig. 7** Initial uniform density (left), 1st (mid) and 6th (right) iterations of our Algorithm to approximate the Markov operator (46) associated to the IFSSDP (41) over [0.049, 1] (top) or the IFSSDP (42) over [0.042, 0.7] (bottom) when probabilities are given by (44)

and second maps are both *increasing*, that is $B = 29.185$ and probabilities $p_i(I)$s defined according to (44). Both figures show the initial uniform density (left panels), the 1st (mid panels) and 6th (right panels) iterations of our Maple algorithm either for the IFSSDP (41) without optimal containment policy (top panels), or for the IFSSDP (42) with optimal containment policy (bottom panels). Note that in the top-left panels of both figures the initial uniform density is given by $\mu_0(I) \equiv \frac{1}{I_3^{st} - I_1^{st}} = \frac{1}{1-0.049} = 1.052$, while in the bottom-left panels of both figures the initial uniform density is given by $\mu_0(I) \equiv \frac{1}{I_3^{st} - I_1^{st}} = \frac{1}{0.7-0.042} = 1.521$.

By comparing Fig. 6b, c and 6e, f with the pairs Figs. 2b, c and 4b, c respectively, or by comparing Figs. 7b, c and 7e, f with the pairs Figs. 3e, f and 5b, c respectively, it is apparent that the addition of a third (lower) map to which it is associated a small probability weight, while leaving the two higher maps almost the same[4] as those in Sects. 2 and 4, does not substantially change the qualitative features of the invariant measure of the disease prevalence. This conclusion equally holds true both in the context of the IFSSDP (6), describing the infection dynamics without containment policy, and of the IFSSDP (19), defining the epidemic dynamics subject to optimal containment. Indeed, we can observe that the pattern of the approximated measures involving only the two higher maps $w_2$ and $w_3$—i.e., excluding the lowest map $w_1$ for values of $I$ larger than 0.1—in Figs. 6 and 7 look quite similar to that in Figs. 2, 3, 4 and 5, where only two maps (shocks) are considered; only the tallest spikes in Figs. 6 and 7 become (quite)

[4] Recall that all parameters' values are kept the same as in (9) and (21), except for the shocks' realizations $r_i$ and the corresponding values of parameters $B$, $\gamma_1$ and $C$ according to Theorem 2.

higher than those in Figs. 2, 3, 4 and 5. Therefore, apart from illustrating the possible consequences of the multiple shock realizations, our two examples with $N = 3$ clearly show that a slight modification of our baseline models discussed in Sects. 2 and 4 does not affect the main characteristics of the invariant measure, at least provided that the additional shock value occurs with a probability that is not too large with respect to the probabilities of the other two shocks. This suggests that our baseline model is robust to slight parameter perturbations and our conclusions regarding the implications of different characteristics of the state-dependent probabilities apply also in more general contexts.

One last point is important to stress at this stage. In order to allow for comparability of our numerical analysis with what we have discussed in the previous sections, we have had to make some specific assumptions regarding the monotonicity properties of the probability functions associated with the different shock values. In particular we have assumed that the probabilities of the two largest shock values are either increasing or decreasing in the number of infectives, such that the probability of the smallest shock value is either decreasing or increasing in disease prevalence, respectively. This clearly is only one of the several possible configurations that probabilities can take across the three different shock realizations. Some other equally plausible configurations may involve the probabilities of the smallest and largest shock values being either increasing or decreasing with prevalence, such that the probability of the intermediate shock value may be either decreasing or increasing with it, respectively. Clearly, in this alternative context analyzing the consequences of the monotonicity properties of the state-dependent probability functions would not make much sense, and their interpretation may be rather limited. Therefore, overall we can conclude that in a multiple shock realizations framework understanding the implications of the features of the state-dependent probabilities on the invariant distribution is more complicated than what we can infer from our two-shock-realizations analysis.

## 7 Conclusion

The ongoing COVID-19 pandemic has brought to light the need to understand the working mechanisms of disease containment policies in order to effectively save human lives and preserve economic conditions. A huge number of works in literature has analyzed from different points of view how the optimal policy should be determined in deterministic settings, but very few have attempted to relate containment policies and stochastic epidemiological dynamics. In this context all the works have assumed that the probability with which shocks affect epidemic dynamics are constant and thus unrelated to disease prevalence. In this paper we contribute to this literature by analyzing the implications of state-dependent probabilities, that is probabilities depending on disease prevalence, for optimal policymaking. We have developed a stylized economic-epidemiological stochastic framework in which random shocks determine the diffusion of a new strain of the disease and the social planner needs to choose the intensity of treatment in order to minimize the social cost of the epidemic management program, accounting for the state-dependency of probabilities. Our results show that in the stochastic steady state complete eradication is never a possible long run outcome where instead disease will always be endemic. Moreover, independently of the features of the state-dependent probabilities, treatment allows to shift leftward the support of the invariant measure, reducing the possible endemic prevalence levels associated with the steady state outcome. However, the features of the state-dependent probabilities are not irrelevant as they affect the shape and spread of disease prevalence over its support, allowing for a steady state outcome characterized by a distribution either highly concentrated over low prevalence levels or more spread out over a larger range of prevalence (possibly higher) levels. Moreover, we characterize the properties of the invariant self-similar measure in terms of singularity and absolutely continuity with respect to the Lebesgue measure, showing that this is ultimately related to the magnitude of the relative magnitude of the net infectivity rate, the extent to which the shock realization affects the net infectivity rate and susceptibles, and the weight attached to potential infections in the objective function. We also extend our baseline model to allow for multiple shock realizations, showing that in this context it is more complicated to assess the implications of the features of the state-dependent probability functions on the invariant distribution because it is no longer possible to isolate the separate role played by each state-dependent probability function.

We are aware that our approach has shortcomings. Indeed, the search for a closed-form solution of the Bellman equation has required us to consider a specific setting and some specific functional forms, which limit the explanatory power of our framework. First, in modeling the dynamics of infectives we have assumed that the same random shock associated with the diffusion of a new disease strain (even if weighted by different parameters) affects both disease incidence and new infections, while in reality it may be more reasonable to model these two effects as driven by different random shocks. Second, in modeling the state-dependency of the probability functions we have assumed that they take some hyperbolic form, while it may be interesting to analyze how different (i.e., linear, quadratic, non-monotonic) specifications of such functions may affect our conclusions. However, both these alternative formulations of disease spreading and state-dependent probabilities would not be compatible with a closed-form characterization of the solution, meaning that the entire analysis would need to be performed numerically. Despite the peculiarities of our model, the closed-form solution of the Bellman equation has allowed us to determine explicitly most of our results, such to rely on a numerical approach only to approximate the steady state invariant distribution. Therefore, we believe that our approach is a quite sensible and informative starting point to discuss the consequences of state-dependent probabilities.

In fact, to the best of our knowledge, ours is the first attempt to introduce state-dependent probabilities in the analysis of the optimal policy in economic-epidemiological frameworks. Therefore, in order to allow for the analytical tractability needed to clarify the main arguments underlying our analysis we have relied on simplifying assumptions limiting the nature of our conclusions. In particular, the abstraction of the epidemic dynamics from the social interactions between infectives and susceptibles has brought us to depart substantially from standard epidemiological models making the comparison of our results with those traditionally discussed in literature particularly complicated. Moreover, we have focused on containment policies taking the form of treatment without exploring how results may change under different types of policies, such as preventive or social distancing measures. Extending our analysis along these directions is currently a priority in our research agenda.

**Acknowledgements** We are indebted to two anonymous referees for their constructive comments on an earlier draft. All remaining errors and omissions are our own sole responsibility. Simone Marsiglio acknowledges financial support from the University of Pisa under the "PRA—Progetti di Ricerca di Ateneo" (Institutional Research Grants)—Project No. PRA_2020_79 "Sustainable development: economic, environmental and social issues". The research of FM is partially supported by NSERC 2019:05237. The usual disclaimer applies.

**Funding** Open access funding provided by Università di Pisa within the CRUI-CARE Agreement.

## Declarations

**Conflict of interest** The authors have no relevant financial or non-financial interests to disclose.

**Open Access** This article is licensed under a Creative Commons Attribution 4.0 International License, which permits use, sharing, adaptation, distribution and reproduction in any medium or format, as long as you give appropriate credit to the original author(s) and the source, provide a link to the Creative Commons licence, and indicate if changes were made. The images or other third party material in this article are included in the article's Creative Commons licence, unless indicated otherwise in a credit line to the material. If material is not included in the article's Creative Commons licence and your intended use is not permitted by statutory regulation or exceeds the permitted use, you will need to obtain permission directly from the copyright holder. To view a copy of this licence, visit http://creativecommons.org/licenses/by/4.0/.

## A Iterated Function Systems

We now briefly review some basic concepts and the main results in the theory of Iterated Function Systems (IFSs) with state-dependent probabilities. The notion of IFS was firstly introduced by Hutchinson (1981) and then extended in different contexts (see Kunze et al. 2012, and the references therein).

The classical definition of IFS consists of a compact metric space $(X, d)$, and a set of $N$ contraction maps on $X$, $\mathbf{w} = \{w_1, \ldots, w_N\}$. Classical results prove that this system has a unique self-similar global attracting set $A$ in $\mathcal{H}(X)$, where $\mathcal{H}(X)$ the space of all compact subsets of $X$ with respect to the *Hausdorff* distance. When an IFS system is equipped with a set of constant probabilities $\mathbf{p} = \{p_1, \ldots, p_N\}$, $\sum_{i=1}^{N} p_i = 1$, then it is also possible to show the existence of a unique self-similar attracting measure $\bar{\mu}$ in the space $\mathcal{M}(X)$ composed by all probability measures on (Borel subsets of) $X$ with respect to the *Monge-Kantorovich* metric.

The family of IFS with state-dependent probabilities extends the above definitions. Within this framework, the probabilities $p_i$ are no longer constant but they are are state-dependent, i.e., $p_i : X \to [0, 1]$ such that:

$$\sum_{i=1}^{N} p_i(x) = 1, \qquad \text{for all } x \in X. \tag{45}$$

The result is an $N$-map *IFS with state-dependent probabilities* (IFSSDP). The Markov operator $M : \mathcal{M}(X) \to \mathcal{M}(X)$ associated with an $N$-map IFSSDP, $(\mathbf{w}, \mathbf{p})$, is defined as:

$$\nu(S) = M\mu(S) = \sum_i \int_{w_i^{-1}(S)} p_i(x)\, d\mu(x), \tag{46}$$

where $\mu \in \mathcal{M}(X)$ and $S \subset X$ is a Borel set.

The following theorem (from results in Elton 1987; and Barnsley et al. 1988) gives conditions as to when an IFSSDP has a unique stationary distribution $\mu$ and the Chaos Game "converges" to $\mu$ in a distributional sense.

**Theorem 3** (Barnsley et al. 1988; Elton 1987) *Suppose that there is a $\delta > 0$ so that $p_i(x) > \delta$ for all $x \in X$ and $i = 1, 2, \ldots, N$ and suppose further that the moduli of continuity of the $p_i$s satisfy Dini's condition (see* Barnsley et al. 1988; Elton 1987*). Then there is a unique stationary distribution $\bar{\mu}$ for the Markov operator. Furthermore, for each continuous function $f : X \to \mathbb{R}$,*

$$\frac{1}{t+1}\sum_{i=0}^{t} f(x_i) \to \int_X f(x)\; d\bar{\mu}(x). \tag{47}$$

Theorem 3 can be used to show the following result.

**Corollary 1** *Suppose that the IFSSDP $\{\mathbf{w}, p_i\}$ satisfies the hypothesis of Theorem 3. Then the support of the invariant measure $\bar{\mu}$ of the $N$-map IFSSDP $(\mathbf{w}, \mathbf{p})$ is the attractor $A$ of the IFS $\mathbf{w}$, i.e.,*

$$\operatorname{supp} \bar{\mu} = A.$$

## B Proof of Theorem 1

First we reduce to the case where the IFS is $\{\alpha x, \beta x + 1 - \beta\}$ acting on [0, 1]. This is possible because for any values of $\alpha, \beta, \tau_1, \tau_2$, the closed interval with endpoints $\frac{\tau_1}{1-\alpha}$ and $\frac{\tau_2}{1-\beta}$ is invariant under the IFS and thus contains the support of $\mu_{\alpha,\beta}$. A simple affine change of variables then gives the IFS $\{\alpha x, \beta x + 1 - \beta\}$ on [0, 1].

(1) The first conclusion is clear since whenever $\alpha + \beta < 1$ the measure $\mu_{\alpha,\beta}$ is supported on a Cantor set with zero Lebesgue measure.
(2) By the results in Ngai and Wang (2005) the IFS $\{\alpha x, \beta x + 1 - \beta\}$ satisfies the transversality condition for all $(\alpha, \beta) \in \Omega$ and then the conclusion follows by Theorem 1.1 in Bárány (2015).
(3) Since we have $\alpha, \beta$ fixed, we use $\mu$ rather than $\mu_{\alpha,\beta}$ to avoid extraneous clutter on our notation. With no loss of generality we assume that $0 < \alpha \le \beta = 1 - \alpha < 1$.

Recall that the "Markov operator" is given by

$$M\nu(S) = \sum_i \int_{w_i^{-1}(S)} p_i(x)\, d\nu(x) = \sum_i \int_S p_i\left(w_i^{-1}(x)\right) d\nu\left(w_i^{-1}(x)\right)$$

and that $\mu$ satisfies $\mu(S) = (M\mu)(S)$. Suppose that $\mu$ is absolutely continuous with density function $f(x)$. Then we obtain the equation

$$\begin{aligned}\int_S f(x)\,dx &= \sum_i \int_{w^{-1}(S)} p_i(y)\, f(y)\, dy \\ &= \int_S \frac{1}{\alpha} p_1(x/\alpha) f(x/\alpha) \chi_{[0,\alpha]}(x) \\ &\quad + \frac{1}{\beta} p_2\left(\frac{x-1+\beta}{\beta}\right) f\left(\frac{x-1+\beta}{\beta}\right) \chi_{[1-\beta,1]}(x)\, dx,\end{aligned}$$

where $\chi_A(x)$ is the characteristic function of the set $A$. For this to be true for all Borel sets $S$ we must have that, for almost every $x$, the two equations

$$f(x) = \frac{1}{\alpha} p_1(x/\alpha) f(x/\alpha), \quad 0 \le x \le \alpha;$$

and

$$f(x) = \frac{1}{\beta} p_2\left(\frac{x-1+\beta}{\beta}\right) f\left(\frac{x-1+\beta}{\beta}\right), \quad \alpha = 1-\beta \le x \le 1.$$

Doing a simple change of variable these become for $0 \le y \le 1$

$$f(\alpha y) = \frac{1}{\alpha} p_1(y) f(y) \quad \Longrightarrow \quad p_1(y) = \frac{\alpha f(\alpha y)}{f(y)} \tag{48}$$

and

$$f(\beta y + 1 - \beta) = \frac{1}{\beta} p_2(y) f(y) \quad \Longrightarrow \quad p_2(y) = \frac{\beta f(\beta y - 1 + \beta)}{f(y)}. \tag{49}$$

Then the condition that $p_1(y) + p_2(y) = 1$ implies that

$$f(y) = \alpha f(\alpha y) + \beta f(\beta + 1 - \beta) \tag{50}$$

for Lebesgue almost every $y \in [0, 1]$. Thus $f(x)$ is a fixed point of the operator

$$T(g)(x) = \alpha g(\alpha y) + \beta g(\beta y + 1 - y).$$

We show that the only fixed point of $T$ which is a density function is the constant function $g(x) = 1$. It is easy to see that $Tg$ is a density if $g$ is a density. Suppose that $g \in C^1[0, 1]$. Then $(Tg)'^2 g'^2 g'(\beta y + 1 - \beta)$ and so (since $t^2 + (1-t)^2 \le \max(t, 1-t)$ for $0 \le t \le 1$) we have $\|(Tg)'\|_\infty \le \beta \|g'\|_\infty$. By induction this means that

$$\left\| \left(T^n g\right)' \right\|_\infty \le \beta^n \left\| g' \right\|_\infty . \tag{51}$$

Next, suppose that we have a density function $g \in C^1[0,1]$ with $\left|g'(x)\right| \leq m$ for all $x \in [0,1]$. Then for all $x \in [0,1]$ we have

$$g(0) - mx \leq g(x) \leq g(0) + mx$$

and thus, integrating over [0, 1], we have

$$g(0) - m/2 \leq 1 \leq g(0) + m/2 \Rightarrow |g(0) - 1| \leq m/2$$

and so

$$|g(x) - 1| \leq mx + m/2 \leq \frac{3}{2}m \Rightarrow \|g - 1\|_\infty \leq \frac{3}{2}m. \tag{52}$$

Next for two functions $f, g \in L^1[0,1]$, integrating the inequality

$$|T(f)(x) - T(g)(x)| \leq \alpha|f(\alpha x) - g(\alpha x)| + \beta|f(\beta x + 1 - \beta) - g(\beta x + 1 - \beta)|$$

over [0, 1] we get

$$\begin{aligned}
&\int_0^1 |T(f)(x) - T(g)(x)|\,dx \\
&\quad\leq \alpha\int_0^1 |f(\alpha x) - g(\alpha x)|\,dx + \beta\int_0^1 |f(\beta x + 1 - \beta) - g(\beta x + 1 - \beta)|\,dx \\
&\quad= \int_0^\alpha |f(u) - g(u)|\,du + \int_\alpha^1 |f(u) - g(u)|\,du = \int_0^1 |f(u) - g(u)|\,du,
\end{aligned}$$

and thus $\|T(f) - T(g)\|_1 \leq \|f - g\|_1$. Let $f$ be a density function and let $\epsilon > 0$ be given. Then there is some density function $g \in C^1[0,1]$ so that $\|f - g\|_1 \leq \epsilon/2$. Then we have

$$\begin{aligned}
\left\|T^n(f) - 1\right\|_1 &\leq \left\|T^n(f) - T^n(g)\right\|_1 + \left\|T^n(g) - 1\right\|_1 \\
&\leq \left\|T^n(f) - T^n(g)\right\|_1 + \left\|T^n(g) - 1\right\|_\infty \\
&\leq \|f - g\|_1 + \frac{3}{2}\left\|\left(T^n g\right)'\right\|_\infty \\
&\leq \|f - g\|_1 + \frac{3\beta^n}{2}\left\|g'\right\|_\infty \leq \epsilon
\end{aligned}$$

for sufficiently large $n$. Thus $T^n f \to 1$ in $L^1[0,1]$ for any density $f$ and so the only density function which satisfies (50) is $f(x) = 1$.

From (48) we get $p_1(x) = \alpha$ as claimed.

## C Proof of Theorem 2

As the RHS in (32) is strictly convex in $y$ whenever the value of constant $A$ is positive ($B$ in (33) is positive as $\gamma_2 < \theta\Omega$ by assumption), to guarantee interiority of the minimum value of $y$ we check the sign of the the derivative with respect to $y$ is negative on the left endpoint of the constraint $0 \leq y \leq \Omega z I + \theta z$ and is positive on its right endpoint; in fact, this is the case:

$$\begin{aligned}\frac{\partial}{\partial y}(RHS) &= -2(\Omega z I + \theta z - y) + 2\beta A B y \sum_{i=1}^{N} a_i r_i^2 \\ &= \begin{cases} -2(\Omega z I + \theta z) < 0 & \text{if } y = 0 \\ 2\beta A B (\Omega z I + \theta z) \sum_{i=1}^{N} a_i r_i^2 > 0 & \text{if } y = \Omega z I + \theta z. \end{cases}\end{aligned}$$

The FOC with respect to $y$ yields the unique solution

$$y^* = \phi z (\Omega I + \theta), \qquad \text{with } \phi = \frac{1}{1 + \beta A B \sum_{i=1}^{N} a_i r_i^2}. \tag{53}$$

Substituting $y^*$ as in (53) into the RHS of (32) after some algebra yields

$$\begin{aligned} V(I, z) &= A z^2 \left(B I^2 + 1\right) + C = A B z^2 I^2 + A z^2 + C \\ &= \gamma_1 z^2 I^2 + \gamma_2 z^2 (1 - I)^2 + [\Omega z I + \theta z - \phi z (\Omega I + \theta)]^2 \\ &\quad + \beta A B \phi^2 z^2 (\Omega I + \theta)^2 \sum_{i=1}^{N} a_i r_i^2 + \beta A \sum_{i=1}^{N} b_i r_i^2 + \beta C \\ &= \left(\gamma_1 + \gamma_2 + \Psi \Omega^2\right) z^2 I^2 + 2(\Psi \theta \Omega - \gamma_2) z^2 I \\ &\quad + \left(\gamma_2 + \Psi \theta^2\right) z^2 + \beta A \sum_{i=1}^{N} b_i r_i^2 + \beta C, \end{aligned}$$

where in the fourth equality we have set $\Psi = (1 - \phi)^2 + \beta A B \phi^2 \sum_{i=1}^{N} a_i r_i^2$.

By equating all similar terms in both sides and setting the coefficient of $z^2 I$ equal to 0 we find that a solution of the Bellman equation (32) is given by the constants $A$, $B$ and $C$ that satisfy

$$\begin{cases} A B = \gamma_1 + \gamma_2 + \Psi \Omega^2 \\ \Psi \theta \Omega - \gamma_2 = 0 \\ A = \gamma_2 + \Psi \theta^2 \\ C = \beta A \sum_{i=1}^{N} b_i r_i^2 + \beta C. \end{cases}$$

From the second equation we get $\Psi = \frac{\gamma_2}{\theta \Omega}$, so that, after substituting this in the third equation, we easily find the value of $A$ as in (35), while, after substituting both values of $\Psi = \frac{\gamma_2}{\theta\Omega}$ and $A$ into the first equation, we have

$$AB = \frac{\Omega+\theta}{\Omega}\gamma_2 B = \gamma_1 + \gamma_2 + \frac{\gamma_2}{\theta}\Omega = \gamma_1 + \frac{\Omega+\theta}{\theta}\gamma_2, \tag{54}$$

which implies that

$$B = \left[\frac{\gamma_1}{(\Omega+\theta)\,\gamma_2} + \frac{1}{\theta}\right]\Omega,$$

which in turn, after substituting $\gamma_1$ with the expression in (34) and after some algebra, yields the expression in (33). Finally, after substituting the value of $A$ as in (35) into the fourth equation one immediately gets the value of $C$ as in (36).

Recalling that $\Psi = (1-\phi)^2 + \beta AB\phi^2 \sum_{i=1}^N a_i r_i^2$ and, from (53), $\phi = \frac{1}{1+\beta AB\sum_{i=1}^N a_i r_i^2}$, the second equation implies that $\gamma_2$ is related to all other parameters according to

$$\begin{aligned}
\gamma_2 = \Psi\theta\Omega &= \left[(1-\phi)^2 + \beta AB\phi^2 \sum_{i=1}^N a_i r_i^2\right]\theta\Omega \\
&= \left[\left(1 - \frac{1}{1+\beta AB\sum_{i=1}^N a_i r_i^2}\right)^2 + \frac{\beta AB\sum_{i=1}^N a_i r_i^2}{\left(1+\beta AB\sum_{i=1}^N a_i r_i^2\right)^2}\right]\theta\Omega \\
&= \frac{\beta AB\sum_{i=1}^N a_i r_i^2}{1+\beta AB\sum_{i=1}^N a_i r_i^2}\theta\Omega \\
&= \frac{\beta\left(\gamma_1 + \frac{\Omega+\theta}{\theta}\gamma_2\right)\sum_{i=1}^N a_i r_i^2}{1+\beta\left(\gamma_1 + \frac{\Omega+\theta}{\theta}\gamma_2\right)\sum_{i=1}^N a_i r_i^2}\theta\Omega,
\end{aligned} \tag{55}$$

where in the last equality we used (54). Note that the last expression requires that $0 < \gamma_2 < \theta\Omega$ must hold.

By further algebraic manipulation, from (55) we get the value of $\gamma_1$ as in (34):

$$\begin{aligned}
&\gamma_2 = \frac{\beta\left(\gamma_1 + \frac{\Omega+\theta}{\theta}\gamma_2\right)\sum_{i=1}^N a_i r_i^2}{1+\beta\left(\gamma_1 + \frac{\Omega+\theta}{\theta}\gamma_2\right)\sum_{i=1}^N a_i r_i^2}\theta\Omega \\
&\iff \quad \gamma_2 + \beta\gamma_2\left(\gamma_1 + \frac{\Omega+\theta}{\theta}\gamma_2\right)\sum_{i=1}^N a_i r_i^2 = \beta\theta\Omega\left(\gamma_1 + \frac{\Omega+\theta}{\theta}\gamma_2\right) \\
&\quad \times \sum_{i=1}^N a_i r_i^2 \\
&\iff \quad \beta\,(\theta\Omega - \gamma_2)\left(\gamma_1 + \frac{\Omega+\theta}{\theta}\gamma_2\right)\sum_{i=1}^N a_i r_i^2 = \gamma_2
\end{aligned}$$

$$\iff \quad \beta\left(\theta\Omega-\gamma_2\right)\gamma_1\sum_{i=1}^{N}a_i r_i^2+\beta\left(\theta\Omega-\gamma_2\right)\frac{\Omega+\theta}{\theta}\gamma_2\sum_{i=1}^{N}a_i r_i^2=\gamma_2$$

$$\iff \quad \beta\left(\theta\Omega-\gamma_2\right)\gamma_1\sum_{i=1}^{N}a_i r_i^2=\left[1-\beta\left(\theta\Omega-\gamma_2\right)\frac{\Omega+\theta}{\theta}\sum_{i=1}^{N}a_i r_i^2\right]\gamma_2$$

$$\iff \gamma_1=\frac{1-\beta\left(\theta\Omega-\gamma_2\right)\frac{\Omega+\theta}{\theta}\sum_{i=1}^{N}a_i r_i^2}{\beta\left(\theta\Omega-\gamma_2\right)\sum_{i=1}^{N}a_i r_i^2}$$

$$\gamma_2=\left[\frac{1}{\beta\left(\theta\Omega-\gamma_2\right)\sum_{i=1}^{N}a_i r_i^2}-\frac{\Omega+\theta}{\theta}\right]\gamma_2.$$

Note that $\gamma_1>0$ because the term in square brackets of the last expression is always strictly positive. In fact, $\gamma_1>0$ is equivalent to

$$\beta\left(\theta\Omega-\gamma_2\right)\left(\Omega+\theta\right)\sum_{i=1}^{N}a_i r_i^2<\theta; \tag{56}$$

noting that $\sum_{i=1}^{N}a_i=1$ together with condition (30) implies

$$\sum_{i=1}^{N}a_i r_i^2<r_N^2\sum_{i=1}^{N}a_i=r_N^2\leq\frac{1}{\left(\eta\tilde{\Omega}\right)^2},$$

and using $\Omega=\eta\tilde{\Omega}-\theta$, the LHS of (56) becomes

$$\begin{aligned}
\beta\left(\theta\Omega-\gamma_2\right)\left(\Omega+\theta\right)\sum_{i=1}^{N}a_i r_i^2 &= \beta\left[\left(\eta\tilde{\Omega}-\theta\right)\theta-\gamma_2\right]\left(\eta\tilde{\Omega}-\theta+\theta\right)\sum_{i=1}^{N}a_i r_i^2\\
&= \beta\left[\left(\eta\tilde{\Omega}-\theta\right)\theta-\gamma_2\right]\eta\tilde{\Omega}\sum_{i=1}^{N}a_i r_i^2\\
&< \beta\left[\left(\eta\tilde{\Omega}-\theta\right)\theta-\gamma_2\right]\frac{\eta\tilde{\Omega}}{\left(\eta\tilde{\Omega}\right)^2}=\frac{\beta\left[\left(\eta\tilde{\Omega}-\theta\right)\theta-\gamma_2\right]}{\eta\tilde{\Omega}},
\end{aligned}$$

so that a sufficient condition for (56) to hold is

$$\begin{aligned}
\frac{\beta\left[\left(\eta\tilde{\Omega}-\theta\right)\theta-\gamma_2\right]}{\eta\tilde{\Omega}}<\theta \quad &\iff \quad \beta\theta\eta\tilde{\Omega}-\beta\theta^2-\beta\gamma_2<\theta\eta\tilde{\Omega}\\
&\iff \quad \left(1-\beta\right)\theta\eta\tilde{\Omega}+\beta\left(\theta^2+\gamma_2\right)>0,
\end{aligned}$$

which definitely holds true as $1-\beta>0$.

By rewriting (55) as

$$\gamma_2 = \frac{\beta\left(\gamma_1 + \frac{\Omega+\theta}{\theta}\gamma_2\right)\sum_{i=1}^N a_i r_i^2}{1+\beta\left(\gamma_1 + \frac{\Omega+\theta}{\theta}\gamma_2\right)\sum_{i=1}^N a_i r_i^2}\theta\Omega$$
$$\Longleftrightarrow \sum_{i=1}^N a_i r_i^2 = \frac{\gamma_2}{\beta\left(\theta\Omega-\gamma_2\right)\left(\gamma_1+\frac{\Omega+\theta}{\theta}\gamma_2\right)},$$

and replacing the value $\sum_{i=1}^N a_i r_i^2$ just obtained into (53) together with the value of the product $AB$ as in (54) easily yields the optimal policy as in (37):

$$y^* = h\left(I, z\right) = \phi z\left(\Omega I + \theta\right) = \frac{z\left(\Omega I+\theta\right)}{1+\beta AB\sum_{i=1}^N a_i r_i^2}$$
$$= \frac{z\left(\Omega I+\theta\right)}{1+\dfrac{\beta\left[\gamma_1+\frac{\Omega+\theta}{\theta}\gamma_2\right]\gamma_2}{\beta\left[\gamma_1+\frac{\Omega+\theta}{\theta}\gamma_2\right]\left(\theta\Omega-\gamma_2\right)}}$$
$$= \frac{z\left(\Omega I+\theta\right)\left(\theta\Omega-\gamma_2\right)}{\theta\Omega-\gamma_2-\gamma_2} = \frac{\theta\Omega-\gamma_2}{\theta\Omega}z\left(\Omega I+\theta\right).$$

Finally, as for each $z \in \{r_1, \cdots, r_N\}$, the value function $V(\cdot, z)$ is defined over the compact interval $[0, 1]$ and continuous, $V(I, z) = ABz^2 I^2 + Az^2 + C$ is bounded over $[0, 1]$; this is enough to apply the standard verification principle and establishes that, in fact, $V(I, z)$ is the value function.

Note that the probabilities defined in (4) and (5) are special cases of the general probabilities defined in (27) of Sect. 6, $p_i(I) = \frac{Ba_i I^2 + b_i}{BI^2+1}$. Specifically, for $i = 1, 2$, the probabilities defined by (4) correspond to (27) when $a_1 = 0$, $a_2 = 1$, $b_1 = 1$ and $b_2 = 0$, while the probabilities defined by (5) correspond to (27) when $a_1 = 1$, $a_2 = 0$, $b_1 = 0$ and $b_2 = 1$. By substituting the proper values for $a_1$, $a_2$, $b_1$ and $b_2$ in the proof above it is straightforward to prove Propositions 1 and 2 as well.
